# The New *Klebsiella* *pneumoniae* ST152 Variants with Hypermucoviscous Phenotype Isolated from Renal Transplant Recipients with Asymptomatic Bacteriuria—Genetic Characteristics by WGS

**DOI:** 10.3390/genes11101189

**Published:** 2020-10-13

**Authors:** Magdalena Wysocka, Roxana Zamudio, Marco R Oggioni, Justyna Gołębiewska, Aleksandra Dudziak, Beata Krawczyk

**Affiliations:** 1 Department of Molecular Biotechnology and Microbiology, Faculty of Chemistry, Gdańsk University of Technology, ul. Narutowicza 11/12, 80-233 Gdańsk, Poland; magwojta@student.pg.edu.pl; 2 Department of Genetics and Genome Biology, University of Leicester, University Road, Leicester LE1 7RH, UK; roxanzamudio@gmail.com (R.Z.); mro5@leicester.ac.uk (M.R.O.); 3 Department of Nephrology, Transplantology and Internal Medicine, Medical University of Gdańsk, ul. Dębinki 7, 80-952 Gdańsk, Poland; jgolebiewska@gumed.edu.pl; 4 Laboratory of Clinical Microbiology, University Centre for Laboratory Diagnostics, Medical University of Gdańsk Clinical Centre, ul. Dębinki 7, 80-952 Gdańsk, Poland; aewiak@uck.gda.pl

**Keywords:** renal transplant, ABU, *Klebsiella pneumoniae*, urinary tract infection, whole genome sequencing, horizontal gene transfer

## Abstract

*Klebsiella pneumoniae* (Kp) is one of the most important etiological factors of urinary tract infections in renal transplant (RTx) recipients. We described the antimicrobial susceptibility phenotypes and genomic features of two hypermucoviscous (HM) Kp isolates recovered from RTx recipients with asymptomatic bacteriuria (ABU). Using whole genome sequencing (WGS) data, we showed that the strains belong to the ST152 lineage with the KL149 capsular serotype, but without *rmp*A/*mag*A genes, which is typical for HM+ hypervirulent Kp. These new strains carried virulence-associated genes that predispose for urinary tract infections (UTIs). Likewise, both strains carried the *ecp* gene encoding pilus common for extended-spectrum β-lactamase (ESBL) *Escherichia coli*. Although the two ST152 isolates were closely related and differed by only nine single nucleotide polymorphisms (SNPs) in their chromosomes, they had different plasmid compositions and chromosomal elements, with isolate KP28872 carrying an ESBL plasmid and an integrative conjugative element. These two isolates are an example of the high plasticity of the *K. pneumoniae* accessory genome. The identification of patients with ABU matched with the correct epidemiological profiling of isolates could facilitate interventions to prevent or rapidly treat *K. pneumoniae* infections.

## 1. Introduction

Urinary tract infections (UTIs) are the most common infectious complication after renal transplantation (RTx), and are reported in up to 80% of patients [1]. Most episodes of UTIs occur during the first six months after transplantation, and one of the most frequently isolated microorganisms is *Klebsiella* spp. [2]. In recent years, the importance of this opportunistic pathogen has increased significantly due to an increase in virulence and resistance to antibiotic therapy [3,4]. *K. pneumoniae* strains were found to be heterogeneous in terms of virulence factors, and recent studies showed that some virulence factors are important for growth at specific tissue sites (lungs, urinary tract, liver, and blood) [5,6,7,8]. 

Hypervirulent *K. pneumoniae* (hvKp) was initially reported in the mid-1980s in Southeast Asia, but similar cases have subsequently been reported worldwide [9]. hvKp has emerged as a clinically significant pathogen associated with highly invasive infections, such as pyogenic liver abscesses [9,10]. Unlike classical *K. pneumoniae* (cKp) strains, approximately half of all hvKp can cause serious community-acquired infections in young, healthy individuals [5]. A defining characteristic of hvKp, is an increased virulence potential associated with a thick hypermucoviscous (HM) capsule and siderophores production [11]. Except for resistance to ampicillin, the majority of HM isolates are rarely resistant to commonly used antibiotics [12], but recent studies reported the acquisition of extended-spectrum β-lactamase (ESBL) and carbapenemase genes [10].

Our attention was drawn to *K. pneumoniae* strains isolated from the urine of two RTx recipients with asymptomatic bacteriuria. The aim of our research was to investigate the genetic background of these strains and compare them phylogenetically with other strains with the same sequence types (STs).

## 2. Materials and Methods 

### 2.1. Patients and Strains

Two RTx recipients hospitalized in the Department of Nephrology, Transplantology, and Internal Medicine at the Medical University of Gdańsk (Poland) were monitored for bacteriuria. All subjects gave their informed consent for inclusion before they participated in the study. Confidential information about the patients was not available to the authors of the publication. Urine cultures were taken at the discretion of the attending physician. The patients suffered from several recurrent episodes of symptomatic UTIs. Case 1 was a type 1 diabetic with recurrent infections with either *K. pneumoniae* MBL+ (metallo-β-lactamase producing) or ESBL+ *K. pneumoniae*, while case 2 was an RTx patient with post-transplant diabetes mellitus and recurrent infections with either *E. coli* (UPEC) or ESBL+ *K. pneumoniae.* Between recurrent infections, the asymptomatic patients were monitored by taking a urine sample followed by a culture in order to retrieve HM *K. pneumoniae.* The HM phenotype was defined as isolates that had the ability to form a viscous string >5 mm in length.

In vitro susceptibility tests of antibiotics were performed using the Vitek-2 (bioMerieux, Polska Sp.zo.o) system following EUCAST v 10.0 (2020) recommendations [13].

### 2.2. Ethical Statements

This study was approved by the ethical committee of the Medical University of Gdańsk (ID: NKBBN/510/2015). 

### 2.3. Molecular Typing of K. pneumoniae Strains by PCR MP Method

The polymerase chain reaction melting profile (PCR MP) procedure was performed as described by Krawczyk et al. [14]. A dendrogram was generated with the Dice Similarity Coefficient (DSC) with a 1% band tolerance setting, and using the unweighted pair group method with arithmetic mean (UPGMA) (FPQuest^TM^ software, BioRad, Ver. 4.5).

### 2.4. Whole Genome Sequencing and Isolates Typing

Genomic DNA was extracted using a DNA extraction kit (BLIRT S.A. Poland), according to the manufacturer’s protocol, and bacterial short-read sequencing was performed using an Illumina HiSeq X 10 platform ((Illumina, Wellcome Trust Sanger, UK) with a 250 bp paired-end protocol (Illumina, location). 

The paired-end short-reads were trimmed using Trimmomatic (Ver. 0.36) [15] and de novo genome assembly was performed using SPAdes (Ver. 3.9.0) [16]). Assembled primary contigs were deposited in the NCBI database under BioProject accession number PRJNA630564 (Table S1). Strain 1 was assigned as isolate KP28872 and strain 2 was assigned as KP28873. Quast (Ver. 5.0.2) [17] was used to generate summary statistics for each assembly. 

In silico MLST (multilocus sequence typing) to identify sequence types (STs) was performed using a Basic Local Alignment Search Tool (BLAST)-based tool [18] on de novo genome assemblies. 

We used the RFPlasmid tool [19] to predict plasmid and chromosomal contigs from draft assemblies. Each contig was assigned a plasmid or chromosomal score. 

The ResFinder [20], VirulenceFinder [21], and PlasmidFinder [22] databases available from the Center for Genomic Epidemiology (http://www.genomicepidemiology.org/) were used to identify and annotate known antimicrobial resistance and virulence factor genes and plasmids. Identified sequences were analyzed using a standalone Basic Local Alignment Search Tool (BLAST) with a cut-off of 95% identity.

The short-reads from our *K. pneumoniae* strains were searched for the presence of the *rmpA* gene. In this analysis, we also included other isolates available from the public database European Nucleotide Archive (ENA): ERR276982 [23], ERR257665, ERR257667, and ERR257678 [24]. In a previous study [25] the presence/absence of the *rmpA* gene was determined in these isolates. Thus, these isolates were our positive and negative control. 

The presence of integrative and conjugative elements ICEKp1 and ICEKp2 was investigated using genes markers as described previously [25]. BLAST was used to identify these gene markers in sequence data from our isolates. 

The Kaptive Web tool was used to identify bacterial surface polysaccharide locus types and evaluate variants [26]. 

A single nucleotide polymorphism (SNP) pairwise difference matrix was obtained using snp-dists (Ver. 0.6.3) [27]. SNPs in the core genome alignment were identified by aligning the short-reads data from each isolate against the reference (GenBank accession no. TOP52_1721_U1) using Snippy (Ver. 3.1) [28]. The analysis pipeline was as follows: paired-end reads of each strain were mapped to the reference genome, variants were identified and annotated, and the effects of variants on genes were predicted using the SnpEff tool (Ver. 4.3t) [29].

To carry out a phylogenetic analysis, we added to our two *K. pneumoniae* genomes, the sequences of 32 published ST152 genomes [6,30,31,32]. Draft genomes were annotated using Prokka (version 1.11) [33] and the pangenome analysis was done using Roary (version 3.6.0) [34] and BLASTp with an 80% identity cut-off. There were 4986 core genes identified and aligned gene-by-gene using Muscle (version 3.8.31) [35], and concatenated using a custom python script. The maximum-likelihood core genome phylogenetic tree was constructed from the concatenated alignment core genes using the general time reversible (GTR) replacement model with four discrete categories of Gamma in RAxML (Ver. 8.2.12) [36]. The ggtree R package (Ver. 1.15.6) [37] was used for visualization, manipulation, and annotation of the phylogenetic trees. The genetic cluster was defined by rhierBAPS [38]. This tool identifies patterns in the genetic data based on the allele frequency; therefore, each cluster has its own allele frequency. In this analysis, we used the aligned sequences as an input, and the maximum number of populations was set to seven. We used a default setting for the other parameters.

## 3. Results

We examined the microbiological and genetic characteristics of two *K. pneumoniae* strains associated with asymptomatic bacteriuria in RTx recipients. Both isolates had a unique HM phenotype based on the string test results. KP28872 had a higher score (150 mm) than KP28873 (35 mm). The HM *K. pneumoniae* KP28873 strain was found to be resistant to amoxicillin/clavulanate, ampicillin, ciprofloxacin, and trimethoprim/sulfamethoxazole, with an intermediate level of piperacillin/tazobactam resistance. Drug susceptibility testing found KP28872 to be ESBL positive. Susceptibility testing of this strain also showed resistance to amoxicillin/clavulanate, ampicillin, cefalotine, cefepime, cefotaxime, ceftazidime, cefuroxime sodium, ciprofloxacin, and piperacillin/tazobactam. 

The strains were isolated within one month. The correlation between strains was determined using the PCR MP genotyping method [14] (Figure S1). The Dice Similarity Coefficient was 61%. 

### 3.1. Genetic Diversity of K. pneumoniae

The two HM *K. pneumoniae* isolates were both assigned to sequence type 152 (Table S1). Antimicrobial resistance- and virulence-associated genes were present in both isolates (Table 1, Tables S2–S4). In accordance with the ESBL phenotypic profile, *bla*_CTX-M-15_ (and *dfrA14*) was present only in isolate KP28872. Both isolates harboured a subset of core chromosomally encoded pathogenicity factors, with enterobactin and salmochelin present in both cases, but yersiniabactin was only in isolate KP28872. Additionally, the genes of capsule type KL149, type 1 fimbriae, type 3 fimbriae, and second type curli were detected for both isolates. The *rmp*A and *rmp*A2 genes typical for the HM phenotype were not found for the tested strains. Both isolates had the same IncFIB and IncFII plasmid replicons, but only KP28872 carried the chromosomal ICEs ICEKp1 and ICEKp2 (Table 1, Tables S2, S5, and S6). We decided to investigate these strains more thoroughly to determine the genetic relatedness between these isolates and identify differences from the other 32 published *Klebsiella pneumoniae* ST152 isolates. 

### 3.2. SNP Diversity of Core Genomes and Phylogenetics

**Figure 1:**
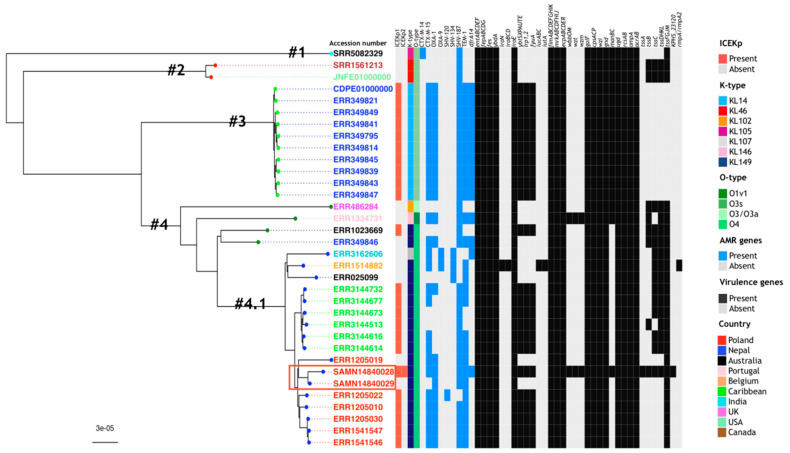
Phylogenetic tree based on the concatenated core genes of the *K. pneumonia* ST152 strains analyzed in this study. Thirty-two ST152 genomes were retrieved from four publications [6,30,31,32], and a core genome was constructed together with the genomes of our isolates: KP28872 (BioSample accession no. SAMN14840028) and KP28873 (BioSample accession no. SAMN14840029) (red box). The rhierBAPS cluster numbers (#1–4.1) are labeled on the phylogenetic tree and the colour of the circle in the external node is linked to their cluster (cyan: cluster no. 1, red: cluster no. 2, light green: cluster no. 3, dark green: cluster no. 4, blue: cluster no. 4.1). The country of origin of the isolates is shown in colour (Poland: red, Nepal: blue, Australia: black, Portugal: pink, Belgium: orange, Caribbean: green, India: cyan, UK: magenta, USA: light green, Canada: brown). In the heatmap, the presence/absence profile of the genotype for genes encoding for resistance determinants (blue: present, grey: absent) and virulence determinants (black: present, grey: absent) is indicated, as well as the presence/absence of ICEKp (red: present, grey: absent), K-type (blue: KL14, red: KL46, orange: KL102, magenta: KL105, grey: KL107, pink: KL146, deep blue: KL149), and O-type (dark green: O1v1, khaki: O3s, light green: O3/O3a, green: O4).

Whole genome comparisons indicated that the two ST152 isolates, despite differences in the accessory genome, were highly related, and showed no core genome single nucleotide polymorphisms (SNPs) and only nine whole genome SNPs when compared to an ST152 reference strain (TOP52_1721_U1). When constructing a core genome phylogenetic tree using whole genome sequences from 32 isolates, KP28872 and KP28873, clustered with a group of six other Polish ST152 genomes [31] (Figure 1). Comparison of KP28872 and KP28873 with the related genomes showed the loss by KP28873 of both the *dfrA14* gene and a neighboring insertion sequence and, in a different contig, of the *bla*_CTX-M-15_ gene from the ISEcp1-*bla*_CTX-M-15_ element [39].

The prevalence of ICEKp1 and ICEKp2 in ST152 *K. pneumoniae* showed that while ICEKp1 was present in multiple isolates, ICEKp2 was present only in the KP28872 (Figure 1). Amongst a group of eight Polish ST152 genomes in the same cluster, two of them had no ICEs. Of the 34 analyzed strains, ICEs did not co-occur with the plasmid carrying mucoid factor encoding genes *rmpA* and *rmpA2* that are associated with increased virulence (Figure 1) [40].

## 4. Discussion

Hypermucoviscous *K. pneumoniae* strains cause infections in immunocompromised patients and individuals with multiple comorbidities and/or diabetes. This report describes two cases of HM Kp isolated from RTx recipients with hospital-acquired ABU. The hypermucoid phenotype of Kp is most often associated with hypervirulent Kp strains, hence our interest in HM Kp strains in this group of patients. 

In our research, we used the PCR MP genotyping method for the bacterial strain differentiation. The genetic typing of the strains suggested a low similarity (Dice Similarity Coefficient 61%) (Figure S1). While the PCR MP method is useful to analyze genetic relatedness [14], the whole genome analysis allowed for the detection of essential elements of HM ABU Kp genomes and their accompanying plasmids [6,8]. 

In contrast to the usual clinical presentation of HM Kp infection, associated with hypervirulent phenotypes [11], both these infections were asymptomatic even though the isolates carried genes for many of the classical virulence factors, including type 1 fimbriae, type 3 fimbrie, curli adhesion, and biofilm formation, as well as siderophores like enterobactin, salmochelin, and, in one case, yersiniabactin. 

The absence of some typical virulence-associated genes like *rmpA*, *rmpA2*, and *magA* was previously shown to confer a non-hypervirulent phenotype to ST152 HM isolates [6]. This indicates that other genes may participate in the regulation of the hypermucoid phenotype, for example, those involved with the regulation of the capsule synthesis genes (*rcsA* and *rcsB*). Increased glucose concentrations lead to the up-regulation of capsule production though *rmpA*, while high extracellular iron concentrations lead to the down-regulation of capsule production [5]. Strain KP28872 was isolated from a patient with type 1 diabetes and it is possible that hyperglycosuria contributed to the HM phenotype (150 mm) seen in this strain. 

So far, only a few cases of antibiotic-resistant hvKp with a HM phenotype have been written in Europe [41,42]. In our study, the WGS analysis revealed several common resistance genes in both isolates and the presence of *bla*_CTX-M-15_ gene for the KP28872 isolate. Both strains had the same IncFIB and IncFII plasmid replicons. IncF plasmids have been shown to mobilize and facilitate the global spread of *bla*_CTX-M-15_ [43]. Chromosomal ICEs, ICEKp1 and ICEKp2 integrative/conjugative elements, were detected only for KP28872. It is alarming that *K. pneumoniae* strains that are highly resistant to antibiotics are increasingly reported, and multi-drug resistant HM Kp isolates may be dangerous pathogens [10,44].

Both our isolates belonged to ST152, which has not previously been described as a high-risk sequence type [45], even though a recent study showed that ST152 could spread between patients [46]. In the collection of genomes analyzed by David and colleagues [31], ST152 is the second most frequent sequence type isolated in Poland, and these six strains tightly grouped with our two isolates. The high plasticity of the *K. pneumoniae* accessory genome is generally referred to the presence or absence of genes and genetic elements [6,7]. It is of note that the only SNPs differing our isolates were in plasmid-located resistance genes and chromosomal islands, which would count as accessory genomes of the species, but also of ST152. This might indicate that the selective pressure on the *K. pneumoniae* accessory genome does not only lead to a high frequency of gain or loss of genes, but also to a higher mutation frequency. Although the hypermucoviscosity phenotype is mainly associated with hvKp strains, we did not find such as high virulence for our isolates in comparison with HM hvKp. However, we observed that there was a noticeable tendency to the acquisition of virulence genes compared with other isolates with type ST152 (Figure 1). The high plasticity of the accessory genome, which allowed rapid import of potential virulence factors by horizontal gene transfer, could indicate the likelihood of this strain evolving to cause more serious disease [47].

Earlier episodes of symptomatic UTIs caused by *E. coli* and ESBL+ *K. pneumoniae* were recorded for the two cases of asymptomatic bacteriuria described in this study. Both experienced urine flow impairment, which is a risk factor for the development of recurrent symptomatic upper UTIs in RTx recipients [4]. The HM phenotype can correlate with a urine flow impairment or difficulty in catheter draining, which could explain the recurrent UTIs in RTx recipients [9,48]. Furthermore, the symptoms of UTIs may be masked due to external immunosuppression in RTx patients. Long-term UTIs or gut colonization of hospital-acquired *K. pneumoniae* strains in immunocompromised patients are also significant risk factors. Symptomatic infection is the most common consequence of bacterial overgrowth and the lack of immunological control of commensal *K. pneumoniae* strains [49]. Gut colonization and bacterial biofilm formation in the urinary tract can favor horizontal gene transfer from uropathogenic strains. HM Kp may compete for the niche and receptor binding with dangerous uropathogenic strains, simultaneously preventing the development of a symptomatic disease.

## 5. Conclusions

*Klebsiella pneumoniae* is a leading cause of health care-associated infections and commonly colonizes hospitalized patients. We report the microbiological and genomic features of the new HM Kp strains of ST152 isolated from RTx recipients with asymptomatic bacteriuria in Europe. The widespread antibiotic resistance and the spread of HM Kp with the tendency to acquire virulence by horizontal gene transfer can lead to the evolution of ABU strains into UTI-causing strains. Hence, we suggest careful microbial monitoring of patients with ABU. On the other hand, different antimicrobial drug susceptibility phenotypes can mask the relatedness of strains, and control of the epidemic situation in the hospital setting can be hampered.

## Figures and Tables

**Table 1:** Genomic characteristics of *K. pneumoniae* ST152 isolates.

	KP28872 (Strain 1)	KP28873 (Strain 2)
Antibiotics	Antimicrobial Resistance Genes	
Aminoglycoside and Fluoroquinolone	*aac(6′)-Ib-cr*	*aac(6’)-Ib-cr*
Aminoglycoside	*aadA1, aadA16, aph(3′)-Ib, aph(6)-Id*	*aadA1, aadA16, aph(3′)-Ib, aph(6)-Id*
Beta-lactam	*bla*_CTX-M-15_ *, bla*_OXA-1_ *, bla*_SHV-187_ *, bla*_TEM-1B_	*bla*_OXA-1_ *, bla*_SHV-187_ *, bla*_TEM-1B_
Fosfomycin	*fosA*	*fosA*
Phenicol	*catA1, catB3*	*catA1, catB3*
Quinolone	*oqxA, oqxB*	*oqxA, oqxB*
Rifampicin	*ARR-3*	*ARR-3*
Sulphonamide	*sul1, sul2*	*sul1, sul2*
Trimethoprim	*dfrA1, dfrA14, dfrA27*	*dfrA1, dfrA27*
ESBL	*bla*_CTX-M 15_ (plasmid)	-
**Virulence-Associated Genetic Elements**	**Virulence-Associated Genes**	
*rmpA* and/or *rmpA2*	-	*-*
Enterobactin	*entABCDEF, fepABCDG, fes, ybdA*	*entABCDEF, fepABCDG, fes, ybdA*
Salmochelin	*iroE*	*iroE*
Yersiniabactin	*ybtAEPSTUX, irp1, irp2, fyuA*	*-*
Fimbriae	*fimABCDEFGHIK, mrkABCDFHIJ, ecpEDCBAR*	*fimABCDEFGHIK, mrkABCDFHIJ, ecpEDCBAR*
**Molecular Data**		
Serotype capsule	KL149	KL149
Plasmid replicon	IncFIB(K) and IncFII(K)	IncFIB(K) and IncFII(K)
ICEs	ICEKp1, ICEKp2	-

**Legend:** ESBL: extended-spectrum-β-lactamase; ICEs: Integrative and Conjugative Elements; *rmp*A and *rmp*A2: regulator of mucoid phenotype A.

## Supplementary Materials

The following are available online: https://www.mdpi.com/2073-4425/11/10/1189/s1. Figure S1: The distance tree of HM Kp isolates using clustering with the unweighted pair group method with arithmetic mean (UPGMA). SAMN14840028: the isolate from case 1 (KP28872); SAMN14840029: the isolate from case 2 (KP28873); Control: ATCC 700603 *K. pneumoniae* reference strain. Table S1: Genome metadata of *Klebsiella pneumoniae* isolates from this study. Table S2: Prediction of plasmid and chromosomal contigs from draft assemblies using the RFPlasmid tool. Table S3: Virulomes of *Klebsiella pneumoniae* isolates from this study detected using the Institut Pasteur MLST databases. Table S4: Antimicrobial resistance genes detected using ResFinder 3.1 from *K. pneumoniae* isolates from this study. Table S5: The presence of integrative and conjugative element ICEKp1 and ICEKp2 in this study. Table S6: Plasmid replicons types identified in the study.
