# Supplementary material for: The New Klebsiella pneumoniae ST152 Variants with Hypermucoviscous Phenotype Isolated from Renal Transplant Recipients with Asymptomatic Bacteriuria—Genetic Characteristics by WGS
Source: Genes (Basel). 2020 Oct 13;11(10):1189. doi: 10.3390/genes11101189 (PMC7601988; doi:10.3390/genes11101189)
Supplement: Supplementary file 1 [file genes-11-01189-s001.pdf]

Article

The new *Klebsiella pneumoniae* ST152 variants with hypermucoviscous phenotype isolated from renal transplant recipients with asymptomatic bacteriuria – genetic characteristics by WGS

Magdalena Wysocka [1], Roxana Zamudio [2], Marco R Oggioni [2], Justyna Gołębiewska [3], Aleksandra Dudziak [4] and Beata Krawczyk [1,*]

[1] Department of Molecular Biotechnology and Microbiology, Faculty of Chemistry, Gdańsk University of Technology, ul. Narutowicza 11/12, 80-233 Gdańsk, Poland.
[2] Department of Genetics and Genome Biology, University of Leicester, University Road, Leicester LE1 7RH, United Kingdom.
[3] Department of Nephrology, Transplantology and Internal Medicine, Medical University of Gdańsk, ul. Dębinki 7, 80-952 Gdańsk, Poland.
[4] Laboratory of Clinical Microbiology, University Centre for Laboratory Diagnostics, Medical University of Gdańsk Clinical Centre, ul. Dębinki 7, 80-952 Gdańsk, Poland.
* Correspondence: beata.krawczyk@pg.edu.pl; Tel.: +48-58-347-23-83

Received: date; Accepted: date; Published: date

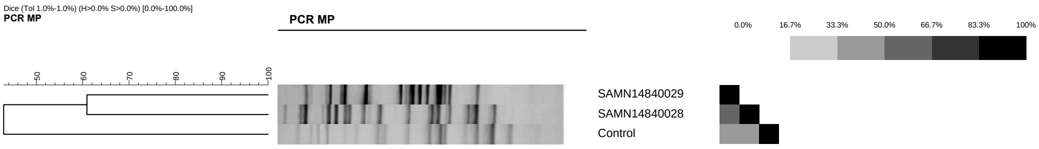

Figure S1: The distance tree of HM Kp isolates using clustering with the unweighted pair group method with arithmetic mean (UPGMA). SAMN14840028 – the isolate from case 1 (KP28872); SAMN14840029 – the isolate from case 2 (KP28873); Control – ATCC 700603 *K. pneumoniae* reference strain.

Table S1: Genome metadata of *Klebsiella pneumoniae* isolates from this study.

Genome name	BioSample	BioProject	Genome accession	Country	Hospital	Year	Source	ST	K type	contigs	Largest contig	Total length	GC (%)	N50	N75	L50	L75	# N's per 100 kbp	num_seqs	Length	Depth	sum_len	min_len	avg_len	max_len	gapP	infB	mdh	pgi	phoE	rpoB	tonB
**KP28872**	SAMN14840028	PRJNA630564	JABFQQ000000000	Poland	UCK Gdansk	2018	urine	152	KL149	365	389262	5928480	56.85	126807	71673	15	30	0.05	1047139	250	49.1	197093943	36	188.2	251	gapA(2)	infB(3)	mdh(2)	pgi(1)	phoE(1)	rpoB(4)	tonB(56)
**KP28873**	SAMN14840029	PRJNA630564	JABFQR000000000	Poland	UCK Gdansk	2018	urine	152	KL149	116	355714	5377974	57.45	139101	84334	14	27	0.00	528831	250	24.8	111070198	36	210	251	gapA(2)	infB(3)	mdh(2)	pgi(1)	phoE(1)	rpoB(4)	tonB(56)

Table S2: Prediction of plasmid and chromosomal contigs from draft assemblies using RFplasmid tool.

Genome name	Case	Contigs	Prediction	Votes chromosomal	Votes plasmid	ContigID
KP28872	case_1	1	chromosomal	0.995	0.005	NODE_1_length_389262_cov_34.9217
KP28872	case_1	10	chromosomal	0.999	0.001	NODE_10_length_174404_cov_36.9282
KP28872	case_1	100	chromosomal	0.716	0.284	NODE_100_length_4052_cov_5.69484
KP28872	case_1	101	chromosomal	0.639	0.361	NODE_101_length_3975_cov_4.62083
KP28872	case_1	102	chromosomal	0.746	0.254	NODE_102_length_3897_cov_4.10759
KP28872	case_1	103	chromosomal	0.794	0.206	NODE_103_length_3894_cov_3.53707
KP28872	case_1	104	chromosomal	0.777	0.223	NODE_104_length_3892_cov_4.82752
KP28872	case_1	105	plasmid	0.122	0.878	NODE_105_length_3827_cov_23.512
KP28872	case_1	106	chromosomal	0.726	0.274	NODE_106_length_3826_cov_4.44732
KP28872	case_1	107	chromosomal	0.758	0.242	NODE_107_length_3823_cov_4.34357
KP28872	case_1	108	chromosomal	0.855	0.145	NODE_108_length_3823_cov_32.9909
KP28872	case_1	109	chromosomal	0.518	0.482	NODE_109_length_3816_cov_3.89917
KP28872	case_1	11	chromosomal	0.999	0.001	NODE_11_length_148322_cov_40.0058
KP28872	case_1	110	chromosomal	0.77	0.23	NODE_110_length_3772_cov_4.08444
KP28872	case_1	111	chromosomal	0.788	0.212	NODE_111_length_3762_cov_3.86893
KP28872	case_1	112	chromosomal	0.908	0.092	NODE_112_length_3761_cov_3.86781
KP28872	case_1	113	chromosomal	0.873	0.127	NODE_113_length_3694_cov_3.98341
KP28872	case_1	114	chromosomal	0.821	0.179	NODE_114_length_3667_cov_3.87103
KP28872	case_1	115	chromosomal	0.652	0.348	NODE_115_length_3648_cov_3.23775
KP28872	case_1	116	plasmid	0.229	0.771	NODE_116_length_3602_cov_15.9452
KP28872	case_1	117	chromosomal	0.661	0.339	NODE_117_length_3443_cov_4.41949
KP28872	case_1	118	chromosomal	0.845	0.155	NODE_118_length_3429_cov_3.51193
KP28872	case_1	119	chromosomal	0.861	0.139	NODE_119_length_3407_cov_5.23363
KP28872	case_1	12	chromosomal	0.996	0.004	NODE_12_length_144887_cov_40.7587
KP28872	case_1	120	chromosomal	0.879	0.121	NODE_120_length_3354_cov_3.80867
KP28872	case_1	121	chromosomal	0.781	0.219	NODE_121_length_3301_cov_29.5639
KP28872	case_1	122	chromosomal	0.879	0.121	NODE_122_length_3202_cov_3.75136
KP28872	case_1	123	chromosomal	0.788	0.212	NODE_123_length_3187_cov_3.33987
KP28872	case_1	124	chromosomal	0.801	0.199	NODE_124_length_3183_cov_4.47778
KP28872	case_1	125	chromosomal	0.682	0.318	NODE_125_length_3140_cov_31.1877
KP28872	case_1	126	chromosomal	0.823	0.177	NODE_126_length_3118_cov_4.14765
KP28872	case_1	127	chromosomal	0.84	0.16	NODE_127_length_3086_cov_5.01263
KP28872	case_1	128	chromosomal	0.753	0.247	NODE_128_length_3080_cov_3.30037
KP28872	case_1	129	chromosomal	0.798	0.202	NODE_129_length_3047_cov_8.93199
KP28872	case_1	13	chromosomal	0.991	0.009	NODE_13_length_143779_cov_35.9103
KP28872	case_1	130	chromosomal	0.613	0.387	NODE_130_length_3021_cov_4.33356
KP28872	case_1	131	chromosomal	0.762	0.238	NODE_131_length_2996_cov_4.62727
KP28872	case_1	132	chromosomal	0.81	0.19	NODE_132_length_2995_cov_4.00754
KP28872	case_1	133	plasmid	0.282	0.718	NODE_133_length_2976_cov_15.4984
KP28872	case_1	134	chromosomal	0.981	0.019	NODE_134_length_2976_cov_371.22
KP28872	case_1	135	chromosomal	0.543	0.457	NODE_135_length_2938_cov_2.94932
KP28872	case_1	136	chromosomal	0.847	0.153	NODE_136_length_2886_cov_4.50338
KP28872	case_1	137	chromosomal	0.788	0.212	NODE_137_length_2875_cov_4.63331
KP28872	case_1	138	chromosomal	0.755	0.245	NODE_138_length_2786_cov_4.811
KP28872	case_1	139	plasmid	0.392	0.608	NODE_139_length_2762_cov_11.4629
KP28872	case_1	14	chromosomal	0.881	0.119	NODE_14_length_131869_cov_35.6086
KP28872	case_1	140	chromosomal	0.64	0.36	NODE_140_length_2760_cov_3.99627
KP28872	case_1	141	chromosomal	0.671	0.329	NODE_141_length_2757_cov_4.50075
KP28872	case_1	142	chromosomal	0.729	0.271	NODE_142_length_2738_cov_3.75272
KP28872	case_1	143	chromosomal	0.736	0.264	NODE_143_length_2713_cov_5.15175
KP28872	case_1	144	chromosomal	0.874	0.126	NODE_144_length_2653_cov_5.18944
KP28872	case_1	145	chromosomal	0.778	0.222	NODE_145_length_2600_cov_5.03409
KP28872	case_1	146	chromosomal	0.645	0.355	NODE_146_length_2565_cov_9.01768

KP28872	case_1	147	chromosomal	0.648	0.352	NODE_147_length_2558_cov_4.57678
KP28872	case_1	148	plasmid	0.476	0.524	NODE_148_length_2511_cov_4.68817
KP28872	case_1	149	chromosomal	0.763	0.237	NODE_149_length_2500_cov_3.35988
KP28872	case_1	15	chromosomal	0.982	0.018	NODE_15_length_126807_cov_40.3171
KP28872	case_1	150	chromosomal	0.795	0.205	NODE_150_length_2491_cov_41.534
KP28872	case_1	151	chromosomal	0.681	0.319	NODE_151_length_2472_cov_3.78455
KP28872	case_1	152	chromosomal	0.657	0.343	NODE_152_length_2446_cov_3.46729
KP28872	case_1	153	chromosomal	0.64	0.36	NODE_153_length_2440_cov_26.7253
KP28872	case_1	154	chromosomal	0.861	0.139	NODE_154_length_2407_cov_5.09056
KP28872	case_1	155	chromosomal	0.676	0.324	NODE_155_length_2394_cov_4.42943
KP28872	case_1	156	chromosomal	0.756	0.244	NODE_156_length_2392_cov_3.29028
KP28872	case_1	157	chromosomal	0.803	0.197	NODE_157_length_2370_cov_2.93371
KP28872	case_1	158	chromosomal	0.6	0.4	NODE_158_length_2365_cov_2.15472
KP28872	case_1	159	chromosomal	0.685	0.315	NODE_159_length_2358_cov_3.56291
KP28872	case_1	16	chromosomal	0.993	0.007	NODE_16_length_119949_cov_35.035
KP28872	case_1	160	chromosomal	0.512	0.488	NODE_160_length_2349_cov_3.65097
KP28872	case_1	161	chromosomal	0.813	0.187	NODE_161_length_2337_cov_3.27168
KP28872	case_1	162	chromosomal	0.83	0.17	NODE_162_length_2316_cov_3.28495
KP28872	case_1	163	chromosomal	0.778	0.222	NODE_163_length_2283_cov_4.06754
KP28872	case_1	164	chromosomal	0.652	0.348	NODE_164_length_2281_cov_2.73775
KP28872	case_1	165	chromosomal	0.823	0.177	NODE_165_length_2277_cov_3.22182
KP28872	case_1	166	chromosomal	0.852	0.148	NODE_166_length_2265_cov_3.73537
KP28872	case_1	167	plasmid	0.491	0.509	NODE_167_length_2263_cov_26.2928
KP28872	case_1	168	chromosomal	0.844	0.156	NODE_168_length_2241_cov_2.79529
KP28872	case_1	169	chromosomal	0.846	0.154	NODE_169_length_2191_cov_4.85525
KP28872	case_1	17	chromosomal	0.969	0.031	NODE_17_length_119920_cov_34.307
KP28872	case_1	170	plasmid	0.466	0.534	NODE_170_length_2180_cov_17.4926
KP28872	case_1	171	plasmid	0.37	0.63	NODE_171_length_2179_cov_3.29876
KP28872	case_1	172	chromosomal	0.565	0.435	NODE_172_length_2177_cov_3.57143
KP28872	case_1	173	chromosomal	0.832	0.168	NODE_173_length_2158_cov_5.97501
KP28872	case_1	174	chromosomal	0.662	0.338	NODE_174_length_2032_cov_3.43478
KP28872	case_1	175	chromosomal	0.712	0.288	NODE_175_length_2023_cov_3.20195
KP28872	case_1	176	chromosomal	0.761	0.239	NODE_176_length_2015_cov_4.20691
KP28872	case_1	177	chromosomal	0.841	0.159	NODE_177_length_1974_cov_4.07696
KP28872	case_1	178	chromosomal	0.733	0.267	NODE_178_length_1946_cov_3.71803
KP28872	case_1	179	chromosomal	0.729	0.271	NODE_179_length_1928_cov_5.37709
KP28872	case_1	18	chromosomal	0.996	0.004	NODE_18_length_105981_cov_40.6289
KP28872	case_1	180	chromosomal	0.79	0.21	NODE_180_length_1924_cov_3.79101
KP28872	case_1	181	chromosomal	0.731	0.269	NODE_181_length_1922_cov_2.92195
KP28872	case_1	182	chromosomal	0.663	0.337	NODE_182_length_1913_cov_261.973
KP28872	case_1	183	chromosomal	0.981	0.019	NODE_183_length_1888_cov_67.3407
KP28872	case_1	184	chromosomal	0.753	0.247	NODE_184_length_1881_cov_4.66463
KP28872	case_1	185	chromosomal	0.799	0.201	NODE_185_length_1874_cov_4.55259
KP28872	case_1	186	chromosomal	0.806	0.194	NODE_186_length_1868_cov_4.31156
KP28872	case_1	187	chromosomal	0.578	0.422	NODE_187_length_1859_cov_2.77385
KP28872	case_1	188	chromosomal	0.675	0.325	NODE_188_length_1805_cov_3.48611
KP28872	case_1	189	plasmid	0.15	0.85	NODE_189_length_1787_cov_15.7918
KP28872	case_1	19	chromosomal	0.629	0.371	NODE_19_length_104776_cov_30.0928
KP28872	case_1	190	chromosomal	0.82	0.18	NODE_190_length_1780_cov_4.36524
KP28872	case_1	191	chromosomal	0.798	0.202	NODE_191_length_1773_cov_8.90212
KP28872	case_1	192	chromosomal	0.789	0.211	NODE_192_length_1763_cov_4.26572
KP28872	case_1	193	chromosomal	0.823	0.177	NODE_193_length_1737_cov_3.98253
KP28872	case_1	194	chromosomal	0.982	0.018	NODE_194_length_1725_cov_369.802
KP28872	case_1	195	chromosomal	0.775	0.225	NODE_195_length_1686_cov_3.3729
KP28872	case_1	196	chromosomal	0.55	0.45	NODE_196_length_1685_cov_3.58022
KP28872	case_1	197	chromosomal	0.787	0.213	NODE_197_length_1652_cov_3.65016

KP28872	case_1	198	chromosomal	0.89	0.11	NODE_198_length_1646_cov_2.79732
KP28872	case_1	199	plasmid	0.291	0.709	NODE_199_length_1632_cov_11.409
KP28872	case_1	2	chromosomal	0.998	0.002	NODE_2_length_292640_cov_38.7891
KP28872	case_1	20	chromosomal	0.996	0.004	NODE_20_length_100509_cov_41.0535
KP28872	case_1	200	chromosomal	0.622	0.378	NODE_200_length_1611_cov_4.04563
KP28872	case_1	201	chromosomal	0.827	0.173	NODE_201_length_1607_cov_9.9366
KP28872	case_1	202	chromosomal	0.837	0.163	NODE_202_length_1590_cov_3.67878
KP28872	case_1	203	plasmid	0.269	0.731	NODE_203_length_1574_cov_29.3273
KP28872	case_1	204	chromosomal	0.748	0.252	NODE_204_length_1532_cov_5.47216
KP28872	case_1	205	chromosomal	0.816	0.184	NODE_205_length_1511_cov_3.16109
KP28872	case_1	206	chromosomal	0.585	0.415	NODE_206_length_1511_cov_14.9331
KP28872	case_1	207	chromosomal	0.745	0.255	NODE_207_length_1471_cov_8.04806
KP28872	case_1	208	chromosomal	0.999	0.001	NODE_208_length_1445_cov_136.04
KP28872	case_1	209	chromosomal	0.515	0.485	NODE_209_length_1436_cov_3.52024
KP28872	case_1	21	chromosomal	0.986	0.014	NODE_21_length_99297_cov_39.5594
KP28872	case_1	210	chromosomal	0.72	0.28	NODE_210_length_1423_cov_3.57727
KP28872	case_1	211	chromosomal	0.569	0.431	NODE_211_length_1412_cov_2.54757
KP28872	case_1	212	chromosomal	0.693	0.307	NODE_212_length_1386_cov_4.0466
KP28872	case_1	213	chromosomal	0.692	0.308	NODE_213_length_1381_cov_3.67331
KP28872	case_1	214	chromosomal	0.769	0.231	NODE_214_length_1367_cov_3.39535
KP28872	case_1	215	plasmid	0.013	0.987	NODE_215_length_1363_cov_29.1042
KP28872	case_1	216	chromosomal	0.833	0.167	NODE_216_length_1361_cov_4.12695
KP28872	case_1	217	chromosomal	0.754	0.246	NODE_217_length_1342_cov_3.56285
KP28872	case_1	218	chromosomal	0.606	0.394	NODE_218_length_1341_cov_2.1606
KP28872	case_1	219	chromosomal	0.746	0.254	NODE_219_length_1335_cov_2.39587
KP28872	case_1	22	chromosomal	0.998	0.002	NODE_22_length_95737_cov_39.6323
KP28872	case_1	220	chromosomal	0.68	0.32	NODE_220_length_1324_cov_2.73857
KP28872	case_1	221	chromosomal	0.587	0.413	NODE_221_length_1310_cov_2.41687
KP28872	case_1	222	chromosomal	0.613	0.387	NODE_222_length_1305_cov_2.53502
KP28872	case_1	223	chromosomal	0.708	0.292	NODE_223_length_1284_cov_3.29577
KP28872	case_1	224	chromosomal	0.843	0.157	NODE_224_length_1279_cov_4.26373
KP28872	case_1	225	chromosomal	0.685	0.315	NODE_225_length_1270_cov_5.8575
KP28872	case_1	226	chromosomal	0.568	0.432	NODE_226_length_1249_cov_3.25597
KP28872	case_1	227	chromosomal	0.804	0.196	NODE_227_length_1243_cov_80.3465
KP28872	case_1	228	chromosomal	0.769	0.231	NODE_228_length_1241_cov_2.4854
KP28872	case_1	229	chromosomal	0.61	0.39	NODE_229_length_1221_cov_5.3243
KP28872	case_1	23	chromosomal	0.999	0.001	NODE_23_length_94236_cov_39.0218
KP28872	case_1	230	plasmid	0.088	0.912	NODE_230_length_1221_cov_406.885
KP28872	case_1	231	chromosomal	0.628	0.372	NODE_231_length_1200_cov_3.58949
KP28872	case_1	232	chromosomal	0.759	0.241	NODE_232_length_1198_cov_2.60125
KP28872	case_1	233	plasmid	0.357	0.643	NODE_233_length_1196_cov_278.592
KP28872	case_1	234	chromosomal	0.605	0.395	NODE_234_length_1195_cov_6.4186
KP28872	case_1	235	chromosomal	0.738	0.262	NODE_235_length_1189_cov_4.45234
KP28872	case_1	236	chromosomal	0.663	0.337	NODE_236_length_1187_cov_4.05135
KP28872	case_1	237	chromosomal	0.805	0.195	NODE_237_length_1187_cov_2.82252
KP28872	case_1	238	chromosomal	0.506	0.494	NODE_238_length_1169_cov_7.61813
KP28872	case_1	239	chromosomal	0.733	0.267	NODE_239_length_1169_cov_4.38004
KP28872	case_1	24	chromosomal	0.991	0.009	NODE_24_length_88599_cov_38.1272
KP28872	case_1	240	chromosomal	0.819	0.181	NODE_240_length_1158_cov_2.57909
KP28872	case_1	241	chromosomal	0.782	0.218	NODE_241_length_1136_cov_3.55902
KP28872	case_1	242	chromosomal	0.65	0.35	NODE_242_length_1134_cov_4.17408
KP28872	case_1	243	chromosomal	0.592	0.408	NODE_243_length_1132_cov_2.16019
KP28872	case_1	244	chromosomal	0.671	0.329	NODE_244_length_1123_cov_3.30688
KP28872	case_1	245	chromosomal	0.614	0.386	NODE_245_length_1118_cov_2.95485
KP28872	case_1	246	chromosomal	0.848	0.152	NODE_246_length_1096_cov_52.2149
KP28872	case_1	247	chromosomal	0.718	0.282	NODE_247_length_1088_cov_3.27102

KP28872	case_1	248	chromosomal	0.711	0.289	NODE_248_length_1086_cov_4.74133
KP28872	case_1	249	chromosomal	0.605	0.395	NODE_249_length_1083_cov_2.47515
KP28872	case_1	25	chromosomal	1	0	NODE_25_length_86606_cov_39.0921
KP28872	case_1	250	chromosomal	0.713	0.287	NODE_250_length_1047_cov_2.72474
KP28872	case_1	251	chromosomal	0.63	0.37	NODE_251_length_1024_cov_4.00211
KP28872	case_1	252	chromosomal	0.996	0.004	NODE_252_length_1013_cov_85.2179
KP28872	case_1	253	chromosomal	0.746	0.254	NODE_253_length_1002_cov_3.30919
KP28872	case_1	254	chromosomal	0.717	0.283	NODE_254_length_1001_cov_2.14394
KP28872	case_1	255	chromosomal	0.723	0.277	NODE_255_length_993_cov_5.41157
KP28872	case_1	256	chromosomal	0.744	0.256	NODE_256_length_992_cov_1.70383
KP28872	case_1	257	chromosomal	0.702	0.298	NODE_257_length_984_cov_3.56009
KP28872	case_1	258	chromosomal	0.656	0.344	NODE_258_length_971_cov_2.56376
KP28872	case_1	259	chromosomal	0.723	0.277	NODE_259_length_936_cov_2.4156
KP28872	case_1	26	chromosomal	0.98	0.02	NODE_26_length_85140_cov_41.6301
KP28872	case_1	260	chromosomal	0.664	0.336	NODE_260_length_932_cov_2.53567
KP28872	case_1	261	chromosomal	0.792	0.208	NODE_261_length_930_cov_4.11723
KP28872	case_1	262	chromosomal	0.76	0.24	NODE_262_length_927_cov_6.07294
KP28872	case_1	263	chromosomal	0.62	0.38	NODE_263_length_915_cov_2.45107
KP28872	case_1	264	chromosomal	0.6	0.4	NODE_264_length_904_cov_2.3821
KP28872	case_1	265	chromosomal	0.626	0.374	NODE_265_length_900_cov_1.78493
KP28872	case_1	266	chromosomal	0.701	0.299	NODE_266_length_894_cov_5.80416
KP28872	case_1	267	chromosomal	0.777	0.223	NODE_267_length_890_cov_7.55474
KP28872	case_1	268	chromosomal	0.745	0.255	NODE_268_length_885_cov_5.6349
KP28872	case_1	269	chromosomal	0.594	0.406	NODE_269_length_885_cov_2.22772
KP28872	case_1	27	chromosomal	0.966	0.034	NODE_27_length_84436_cov_34.0821
KP28872	case_1	270	chromosomal	0.714	0.286	NODE_270_length_848_cov_2.89883
KP28872	case_1	271	chromosomal	0.596	0.404	NODE_271_length_821_cov_1.82661
KP28872	case_1	272	chromosomal	0.687	0.313	NODE_272_length_820_cov_4.68102
KP28872	case_1	273	plasmid	0	1	NODE_273_length_820_cov_145.094
KP28872	case_1	274	chromosomal	0.691	0.309	NODE_274_length_818_cov_3.00405
KP28872	case_1	275	chromosomal	0.621	0.379	NODE_275_length_798_cov_2.01664
KP28872	case_1	276	chromosomal	0.707	0.293	NODE_276_length_791_cov_4.34734
KP28872	case_1	277	chromosomal	0.591	0.409	NODE_277_length_775_cov_2.47421
KP28872	case_1	278	chromosomal	0.948	0.052	NODE_278_length_757_cov_141.529
KP28872	case_1	279	chromosomal	0.64	0.36	NODE_279_length_754_cov_1.9099
KP28872	case_1	28	chromosomal	0.959	0.041	NODE_28_length_77896_cov_39.5339
KP28872	case_1	280	chromosomal	0.705	0.295	NODE_280_length_744_cov_4.69565
KP28872	case_1	281	chromosomal	0.724	0.276	NODE_281_length_741_cov_2.61145
KP28872	case_1	282	chromosomal	0.567	0.433	NODE_282_length_740_cov_2.03017
KP28872	case_1	283	chromosomal	0.652	0.348	NODE_283_length_727_cov_31.3
KP28872	case_1	284	chromosomal	0.719	0.281	NODE_284_length_715_cov_3.05172
KP28872	case_1	285	chromosomal	0.571	0.429	NODE_285_length_715_cov_2.26019
KP28872	case_1	286	chromosomal	0.722	0.278	NODE_286_length_708_cov_3.27575
KP28872	case_1	287	chromosomal	0.586	0.414	NODE_287_length_700_cov_2.76244
KP28872	case_1	288	plasmid	0.5	0.5	NODE_288_length_683_cov_0.942244
KP28872	case_1	289	chromosomal	0.708	0.292	NODE_289_length_682_cov_1.20661
KP28872	case_1	29	chromosomal	0.981	0.019	NODE_29_length_76370_cov_41.0225
KP28872	case_1	290	chromosomal	0.68	0.32	NODE_290_length_669_cov_1.36824
KP28872	case_1	291	chromosomal	0.647	0.353	NODE_291_length_669_cov_40.9949
KP28872	case_1	292	chromosomal	0.629	0.371	NODE_292_length_668_cov_2.16244
KP28872	case_1	293	chromosomal	0.639	0.361	NODE_293_length_666_cov_2.71986
KP28872	case_1	294	chromosomal	0.621	0.379	NODE_294_length_664_cov_2.30153
KP28872	case_1	295	chromosomal	0.601	0.399	NODE_295_length_664_cov_1.65247
KP28872	case_1	296	chromosomal	0.552	0.448	NODE_296_length_643_cov_3.37809
KP28872	case_1	297	chromosomal	0.73	0.27	NODE_297_length_632_cov_2.66486
KP28872	case_1	298	chromosomal	0.599	0.401	NODE_298_length_625_cov_2.48723

KP28872	case_1	299	chromosomal	0.514	0.486	NODE_299_length_621_cov_4.38051
KP28872	case_1	3	chromosomal	1	0	NODE_3_length_260172_cov_39.2366
KP28872	case_1	30	chromosomal	0.962	0.038	NODE_30_length_71673_cov_98.3973
KP28872	case_1	300	chromosomal	0.508	0.492	NODE_300_length_619_cov_2.56458
KP28872	case_1	301	chromosomal	0.861	0.139	NODE_301_length_610_cov_78.94
KP28872	case_1	302	chromosomal	0.599	0.401	NODE_302_length_609_cov_2.67857
KP28872	case_1	303	chromosomal	0.557	0.443	NODE_303_length_609_cov_2.17857
KP28872	case_1	304	plasmid	0.474	0.526	NODE_304_length_607_cov_48.9057
KP28872	case_1	305	chromosomal	0.639	0.361	NODE_305_length_585_cov_3.99016
KP28872	case_1	306	chromosomal	0.756	0.244	NODE_306_length_558_cov_3.24948
KP28872	case_1	307	chromosomal	0.765	0.235	NODE_307_length_528_cov_1.62749
KP28872	case_1	308	chromosomal	0.617	0.383	NODE_308_length_518_cov_1.26077
KP28872	case_1	309	chromosomal	0.66	0.34	NODE_309_length_508_cov_2.96752
KP28872	case_1	31	chromosomal	0.959	0.041	NODE_31_length_62746_cov_96.3854
KP28872	case_1	310	chromosomal	0.66	0.34	NODE_310_length_506_cov_2.04895
KP28872	case_1	311	chromosomal	0.671	0.329	NODE_311_length_505_cov_3.07944
KP28872	case_1	312	plasmid	0.099	0.901	NODE_312_length_491_cov_90.2609
KP28872	case_1	313	chromosomal	0.576	0.424	NODE_313_length_488_cov_3.29197
KP28872	case_1	314	chromosomal	0.663	0.337	NODE_314_length_479_cov_24.3333
KP28872	case_1	315	chromosomal	0.611	0.389	NODE_315_length_462_cov_1.29091
KP28872	case_1	316	chromosomal	0.668	0.332	NODE_316_length_454_cov_1.76127
KP28872	case_1	317	chromosomal	0.688	0.312	NODE_317_length_447_cov_3.09459
KP28872	case_1	318	chromosomal	0.559	0.441	NODE_318_length_445_cov_1.20924
KP28872	case_1	319	chromosomal	0.606	0.394	NODE_319_length_436_cov_2.8468
KP28872	case_1	32	chromosomal	0.987	0.013	NODE_32_length_58974_cov_37.9615
KP28872	case_1	320	chromosomal	0.623	0.377	NODE_320_length_436_cov_2.14485
KP28872	case_1	321	chromosomal	0.625	0.375	NODE_321_length_428_cov_1.66382
KP28872	case_1	322	chromosomal	0.617	0.383	NODE_322_length_427_cov_2.62571
KP28872	case_1	323	chromosomal	0.682	0.318	NODE_323_length_422_cov_2.51304
KP28872	case_1	324	chromosomal	0.715	0.285	NODE_324_length_418_cov_4.22287
KP28872	case_1	325	chromosomal	0.574	0.426	NODE_325_length_405_cov_3.60671
KP28872	case_1	326	chromosomal	0.661	0.339	NODE_326_length_395_cov_0.981132
KP28872	case_1	327	chromosomal	0.679	0.321	NODE_327_length_394_cov_0.974763
KP28872	case_1	328	chromosomal	0.65	0.35	NODE_328_length_386_cov_30.9256
KP28872	case_1	329	chromosomal	0.564	0.436	NODE_329_length_385_cov_0.987013
KP28872	case_1	33	chromosomal	0.907	0.093	NODE_33_length_50217_cov_28.9361
KP28872	case_1	330	chromosomal	0.642	0.358	NODE_330_length_381_cov_1.06908
KP28872	case_1	331	chromosomal	0.712	0.288	NODE_331_length_380_cov_0.953795
KP28872	case_1	332	chromosomal	0.677	0.323	NODE_332_length_380_cov_2.28383
KP28872	case_1	333	chromosomal	0.642	0.358	NODE_333_length_377_cov_0.986667
KP28872	case_1	334	chromosomal	0.658	0.342	NODE_334_length_374_cov_1.6431
KP28872	case_1	335	chromosomal	0.618	0.382	NODE_335_length_374_cov_3.61616
KP28872	case_1	336	chromosomal	0.642	0.358	NODE_336_length_373_cov_1.01351
KP28872	case_1	337	chromosomal	0.687	0.313	NODE_337_length_373_cov_3.30405
KP28872	case_1	338	chromosomal	0.668	0.332	NODE_338_length_368_cov_1.51546
KP28872	case_1	339	chromosomal	0.682	0.318	NODE_339_length_368_cov_1.34708
KP28872	case_1	34	plasmid	0.038	0.962	NODE_34_length_45797_cov_21.1749
KP28872	case_1	340	chromosomal	0.597	0.403	NODE_340_length_367_cov_0.975862
KP28872	case_1	341	chromosomal	0.559	0.441	NODE_341_length_366_cov_2.06574
KP28872	case_1	342	chromosomal	0.613	0.387	NODE_342_length_360_cov_1.08127
KP28872	case_1	343	chromosomal	0.659	0.341	NODE_343_length_359_cov_2.20922
KP28872	case_1	344	chromosomal	0.597	0.403	NODE_344_length_358_cov_1.13167
KP28872	case_1	345	chromosomal	0.644	0.356	NODE_345_length_358_cov_1.32028
KP28872	case_1	346	chromosomal	0.695	0.305	NODE_346_length_357_cov_1.81071
KP28872	case_1	347	chromosomal	0.597	0.403	NODE_347_length_357_cov_0.985714
KP28872	case_1	348	chromosomal	0.677	0.323	NODE_348_length_356_cov_2.17921

KP28872	case_1	349	chromosomal	0.686	0.314	NODE_349_length_352_cov_2.09455
KP28872	case_1	35	plasmid	0.019	0.981	NODE_35_length_42433_cov_19.7452
KP28872	case_1	350	chromosomal	0.659	0.341	NODE_350_length_351_cov_2.08394
KP28872	case_1	351	chromosomal	0.613	0.387	NODE_351_length_350_cov_1.85348
KP28872	case_1	352	chromosomal	0.622	0.378	NODE_352_length_348_cov_0.9631
KP28872	case_1	353	chromosomal	0.634	0.366	NODE_353_length_346_cov_3.5948
KP28872	case_1	354	chromosomal	0.622	0.378	NODE_354_length_345_cov_1.08209
KP28872	case_1	355	chromosomal	0.578	0.422	NODE_355_length_344_cov_1.01873
KP28872	case_1	356	chromosomal	0.678	0.322	NODE_356_length_343_cov_2.13534
KP28872	case_1	357	chromosomal	0.623	0.377	NODE_357_length_338_cov_1.2069
KP28872	case_1	358	chromosomal	0.842	0.158	NODE_358_length_338_cov_145.402
KP28872	case_1	359	chromosomal	0.682	0.318	NODE_359_length_336_cov_1.0888
KP28872	case_1	36	chromosomal	0.975	0.025	NODE_36_length_40905_cov_37.1967
KP28872	case_1	360	plasmid	0.294	0.706	NODE_360_length_332_cov_27.4275
KP28872	case_1	361	chromosomal	0.689	0.311	NODE_361_length_303_cov_40.4027
KP28872	case_1	362	chromosomal	0.585	0.415	NODE_362_length_217_cov_38.4714
KP28872	case_1	363	chromosomal	0.724	0.276	NODE_363_length_207_cov_92.3923
KP28872	case_1	364	chromosomal	0.715	0.285	NODE_364_length_202_cov_48.192
KP28872	case_1	365	chromosomal	0.744	0.256	NODE_365_length_201_cov_32.6452
KP28872	case_1	366	chromosomal	0.653	0.347	NODE_366_length_197_cov_56.5833
KP28872	case_1	367	chromosomal	0.582	0.418	NODE_367_length_188_cov_72.2973
KP28872	case_1	368	chromosomal	0.641	0.359	NODE_368_length_188_cov_49.2793
KP28872	case_1	369	chromosomal	0.578	0.422	NODE_369_length_186_cov_34.8257
KP28872	case_1	37	plasmid	0.133	0.867	NODE_37_length_37136_cov_21.0137
KP28872	case_1	370	chromosomal	0.71	0.29	NODE_370_length_182_cov_99.4476
KP28872	case_1	371	chromosomal	0.615	0.385	NODE_371_length_177_cov_66.07
KP28872	case_1	372	chromosomal	0.637	0.363	NODE_372_length_176_cov_26.3131
KP28872	case_1	373	chromosomal	0.617	0.383	NODE_373_length_157_cov_42.575
KP28872	case_1	374	chromosomal	0.716	0.284	NODE_374_length_156_cov_107.114
KP28872	case_1	375	chromosomal	0.71	0.29	NODE_375_length_156_cov_177.709
KP28872	case_1	376	chromosomal	0.588	0.412	NODE_376_length_152_cov_40.2933
KP28872	case_1	377	chromosomal	0.608	0.392	NODE_377_length_152_cov_24
KP28872	case_1	378	chromosomal	0.62	0.38	NODE_378_length_142_cov_84.4154
KP28872	case_1	379	chromosomal	0.602	0.398	NODE_379_length_121_cov_173.364
KP28872	case_1	38	chromosomal	0.997	0.003	NODE_38_length_34549_cov_40.0918
KP28872	case_1	380	chromosomal	0.751	0.249	NODE_380_length_120_cov_1.62791
KP28872	case_1	381	chromosomal	0.751	0.249	NODE_381_length_120_cov_30.3721
KP28872	case_1	382	chromosomal	0.668	0.332	NODE_382_length_109_cov_42.2812
KP28872	case_1	383	chromosomal	0.615	0.385	NODE_383_length_101_cov_117.625
KP28872	case_1	384	chromosomal	0.632	0.368	NODE_384_length_96_cov_105.368
KP28872	case_1	385	chromosomal	0.649	0.351	NODE_385_length_89_cov_32.6667
KP28872	case_1	386	chromosomal	0.627	0.373	NODE_386_length_87_cov_41.3
KP28872	case_1	387	chromosomal	0.675	0.325	NODE_387_length_85_cov_39
KP28872	case_1	388	chromosomal	0.772	0.228	NODE_388_length_85_cov_329.5
KP28872	case_1	389	chromosomal	0.66	0.34	NODE_389_length_79_cov_181.5
KP28872	case_1	39	chromosomal	0.77	0.23	NODE_39_length_34472_cov_31.157
KP28872	case_1	390	chromosomal	0.847	0.153	NODE_390_length_78_cov_76
KP28872	case_1	391	chromosomal	0.583	0.417	NODE_391_length_78_cov_121
KP28872	case_1	4	chromosomal	1	0	NODE_4_length_230310_cov_35.9925
KP28872	case_1	40	chromosomal	0.909	0.091	NODE_40_length_33913_cov_38.9698
KP28872	case_1	41	chromosomal	0.646	0.354	NODE_41_length_31841_cov_27.2008
KP28872	case_1	42	chromosomal	0.932	0.068	NODE_42_length_29764_cov_38.9923
KP28872	case_1	43	chromosomal	0.944	0.056	NODE_43_length_28468_cov_34.8087
KP28872	case_1	44	chromosomal	0.994	0.006	NODE_44_length_26346_cov_38.1109
KP28872	case_1	45	chromosomal	0.771	0.229	NODE_45_length_23398_cov_27.2035
KP28872	case_1	46	chromosomal	0.665	0.335	NODE_46_length_23195_cov_39.9896

KP28872	case_1	47	chromosomal	0.574	0.426	NODE_47_length_22463_cov_32.9732
KP28872	case_1	48	chromosomal	0.952	0.048	NODE_48_length_20303_cov_42.5318
KP28872	case_1	49	chromosomal	0.906	0.094	NODE_49_length_19579_cov_39.8777
KP28872	case_1	5	chromosomal	0.995	0.005	NODE_5_length_228449_cov_35.4333
KP28872	case_1	50	chromosomal	0.547	0.453	NODE_50_length_19359_cov_4.40763
KP28872	case_1	51	chromosomal	0.867	0.133	NODE_51_length_19046_cov_43.0785
KP28872	case_1	52	plasmid	0.009	0.991	NODE_52_length_16943_cov_14.2677
KP28872	case_1	53	chromosomal	0.77	0.23	NODE_53_length_15431_cov_4.48815
KP28872	case_1	54	plasmid	0.436	0.564	NODE_54_length_14967_cov_35.5692
KP28872	case_1	55	chromosomal	0.831	0.169	NODE_55_length_14763_cov_38.155
KP28872	case_1	56	chromosomal	0.901	0.099	NODE_56_length_13828_cov_37.5022
KP28872	case_1	57	chromosomal	0.895	0.105	NODE_57_length_13789_cov_32.8689
KP28872	case_1	58	plasmid	0.103	0.897	NODE_58_length_13687_cov_13.536
KP28872	case_1	59	plasmid	0.081	0.919	NODE_59_length_11614_cov_21.6892
KP28872	case_1	6	chromosomal	0.998	0.002	NODE_6_length_203863_cov_36.4848
KP28872	case_1	60	chromosomal	0.864	0.136	NODE_60_length_10594_cov_4.70533
KP28872	case_1	61	chromosomal	0.728	0.272	NODE_61_length_10396_cov_4.66276
KP28872	case_1	62	plasmid	0.43	0.57	NODE_62_length_10267_cov_4.3949
KP28872	case_1	63	chromosomal	0.911	0.089	NODE_63_length_9588_cov_33.8295
KP28872	case_1	64	chromosomal	0.724	0.276	NODE_64_length_8827_cov_4.46914
KP28872	case_1	65	chromosomal	0.737	0.263	NODE_65_length_8825_cov_4.01235
KP28872	case_1	66	chromosomal	0.83	0.17	NODE_66_length_8614_cov_4.06958
KP28872	case_1	67	chromosomal	0.722	0.278	NODE_67_length_7982_cov_37.0061
KP28872	case_1	68	chromosomal	0.857	0.143	NODE_68_length_7826_cov_34.0514
KP28872	case_1	69	chromosomal	0.832	0.168	NODE_69_length_7821_cov_4.72314
KP28872	case_1	7	chromosomal	0.996	0.004	NODE_7_length_203049_cov_34.8131
KP28872	case_1	70	plasmid	0.202	0.798	NODE_70_length_7688_cov_20.5504
KP28872	case_1	71	plasmid	0.443	0.557	NODE_71_length_7445_cov_3.36767
KP28872	case_1	72	chromosomal	0.882	0.118	NODE_72_length_7312_cov_4.60705
KP28872	case_1	73	chromosomal	0.691	0.309	NODE_73_length_6967_cov_5.51466
KP28872	case_1	74	plasmid	0.052	0.948	NODE_74_length_6916_cov_24.628
KP28872	case_1	75	chromosomal	0.673	0.327	NODE_75_length_6686_cov_6.00999
KP28872	case_1	76	plasmid	0.042	0.958	NODE_76_length_6551_cov_10.9166
KP28872	case_1	77	plasmid	0.239	0.761	NODE_77_length_6167_cov_22.7044
KP28872	case_1	78	chromosomal	0.591	0.409	NODE_78_length_5964_cov_25.4296
KP28872	case_1	79	plasmid	0.224	0.776	NODE_79_length_5859_cov_25.647
KP28872	case_1	8	chromosomal	0.993	0.007	NODE_8_length_190620_cov_41.9811
KP28872	case_1	80	chromosomal	0.88	0.12	NODE_80_length_5449_cov_4.15004
KP28872	case_1	81	chromosomal	0.815	0.185	NODE_81_length_5391_cov_3.85566
KP28872	case_1	82	chromosomal	0.893	0.107	NODE_82_length_5263_cov_4.15947
KP28872	case_1	83	chromosomal	0.898	0.102	NODE_83_length_5250_cov_4.607
KP28872	case_1	84	plasmid	0.037	0.963	NODE_84_length_5212_cov_29.0715
KP28872	case_1	85	plasmid	0.324	0.676	NODE_85_length_5208_cov_3.69577
KP28872	case_1	86	chromosomal	0.925	0.075	NODE_86_length_5160_cov_4.06532
KP28872	case_1	87	chromosomal	0.753	0.247	NODE_87_length_4901_cov_4.31219
KP28872	case_1	88	plasmid	0.36	0.64	NODE_88_length_4782_cov_3.88842
KP28872	case_1	89	plasmid	0.14	0.86	NODE_89_length_4667_cov_12.5203
KP28872	case_1	9	chromosomal	0.996	0.004	NODE_9_length_177480_cov_42.0453
KP28872	case_1	90	chromosomal	0.636	0.364	NODE_90_length_4654_cov_3.92659
KP28872	case_1	91	chromosomal	0.605	0.395	NODE_91_length_4635_cov_4.72159
KP28872	case_1	92	chromosomal	0.85	0.15	NODE_92_length_4621_cov_5.67826
KP28872	case_1	93	plasmid	0.475	0.525	NODE_93_length_4506_cov_19.8607
KP28872	case_1	94	chromosomal	0.722	0.278	NODE_94_length_4482_cov_3.9807
KP28872	case_1	95	chromosomal	0.763	0.237	NODE_95_length_4393_cov_4.24351
KP28872	case_1	96	chromosomal	0.747	0.253	NODE_96_length_4307_cov_26.1305
KP28872	case_1	97	chromosomal	0.844	0.156	NODE_97_length_4213_cov_4.23042

KP28872	case_1	98	chromosomal	0.811	0.189	NODE_98_length_4107_cov_3.78983
KP28872	case_1	99	chromosomal	0.814	0.186	NODE_99_length_4104_cov_3.77204
KP28873	case_2	1	chromosomal	1	0	NODE_1_length_355714_cov_24.3492
KP28873	case_2	10	chromosomal	0.99	0.01	NODE_10_length_159341_cov_22.3163
KP28873	case_2	100	chromosomal	0.606	0.394	NODE_100_length_557_cov_19.8833
KP28873	case_2	101	chromosomal	0.663	0.337	NODE_101_length_479_cov_18.2313
KP28873	case_2	102	chromosomal	0.888	0.112	NODE_102_length_447_cov_80.5811
KP28873	case_2	103	chromosomal	0.609	0.391	NODE_103_length_433_cov_34.0197
KP28873	case_2	104	chromosomal	0.65	0.35	NODE_104_length_386_cov_23.89
KP28873	case_2	105	chromosomal	0.673	0.327	NODE_105_length_365_cov_19.0278
KP28873	case_2	106	chromosomal	0.876	0.124	NODE_106_length_338_cov_100.479
KP28873	case_2	107	chromosomal	0.586	0.414	NODE_107_length_333_cov_51.2734
KP28873	case_2	108	chromosomal	0.688	0.312	NODE_108_length_301_cov_28.8348
KP28873	case_2	109	plasmid	0.497	0.503	NODE_109_length_291_cov_13.3178
KP28873	case_2	11	chromosomal	0.988	0.012	NODE_11_length_156887_cov_20.8155
KP28873	case_2	110	plasmid	0.275	0.725	NODE_110_length_277_cov_12.07
KP28873	case_2	111	chromosomal	0.709	0.291	NODE_111_length_269_cov_22.9531
KP28873	case_2	112	chromosomal	0.768	0.232	NODE_112_length_251_cov_41.092
KP28873	case_2	113	chromosomal	0.645	0.355	NODE_113_length_241_cov_20.1159
KP28873	case_2	114	chromosomal	0.764	0.236	NODE_114_length_215_cov_25.7609
KP28873	case_2	115	chromosomal	0.724	0.276	NODE_115_length_207_cov_20.0692
KP28873	case_2	116	chromosomal	0.634	0.366	NODE_116_length_205_cov_22.1172
KP28873	case_2	117	chromosomal	0.622	0.378	NODE_117_length_195_cov_33.1102
KP28873	case_2	118	chromosomal	0.585	0.415	NODE_118_length_195_cov_81.0513
KP28873	case_2	119	chromosomal	0.627	0.373	NODE_119_length_193_cov_17.3276
KP28873	case_2	12	chromosomal	0.999	0.001	NODE_12_length_148201_cov_25.4964
KP28873	case_2	120	chromosomal	0.553	0.447	NODE_120_length_188_cov_20.027
KP28873	case_2	121	chromosomal	0.62	0.38	NODE_121_length_185_cov_26.4722
KP28873	case_2	122	chromosomal	0.625	0.375	NODE_122_length_183_cov_28.2547
KP28873	case_2	123	chromosomal	0.586	0.414	NODE_123_length_179_cov_42.1667
KP28873	case_2	124	chromosomal	0.654	0.346	NODE_124_length_169_cov_26.4457
KP28873	case_2	125	plasmid	0.48	0.52	NODE_125_length_153_cov_10.6711
KP28873	case_2	126	chromosomal	0.568	0.432	NODE_126_length_150_cov_18.7123
KP28873	case_2	127	chromosomal	0.62	0.38	NODE_127_length_133_cov_37.6
KP28873	case_2	128	chromosomal	0.607	0.393	NODE_128_length_128_cov_50.6471
KP28873	case_2	129	chromosomal	0.735	0.265	NODE_129_length_120_cov_24.8372
KP28873	case_2	13	chromosomal	0.996	0.004	NODE_13_length_144891_cov_25.4965
KP28873	case_2	130	plasmid	0.242	0.758	NODE_130_length_118_cov_14.1951
KP28873	case_2	131	plasmid	0.253	0.747	NODE_131_length_114_cov_13.7568
KP28873	case_2	132	chromosomal	0.619	0.381	NODE_132_length_107_cov_36.9333
KP28873	case_2	133	chromosomal	0.618	0.382	NODE_133_length_104_cov_29.8889
KP28873	case_2	134	chromosomal	0.615	0.385	NODE_134_length_101_cov_88.7083
KP28873	case_2	135	chromosomal	0.586	0.414	NODE_135_length_96_cov_17.5263
KP28873	case_2	136	plasmid	0.371	0.629	NODE_136_length_95_cov_25.8889
KP28873	case_2	137	chromosomal	0.636	0.364	NODE_137_length_94_cov_20.4706
KP28873	case_2	138	plasmid	0.198	0.802	NODE_138_length_92_cov_13.6
KP28873	case_2	139	chromosomal	0.649	0.351	NODE_139_length_89_cov_25.75
KP28873	case_2	14	chromosomal	0.99	0.01	NODE_14_length_139101_cov_22.6223
KP28873	case_2	140	chromosomal	0.623	0.377	NODE_140_length_88_cov_54.2727
KP28873	case_2	141	chromosomal	0.772	0.228	NODE_141_length_85_cov_77.75
KP28873	case_2	142	plasmid	0.239	0.761	NODE_142_length_81_cov_12
KP28873	case_2	143	chromosomal	0.657	0.343	NODE_143_length_81_cov_68.75
KP28873	case_2	144	chromosomal	0.573	0.427	NODE_144_length_79_cov_48
KP28873	case_2	145	chromosomal	0.744	0.256	NODE_145_length_79_cov_46
KP28873	case_2	146	chromosomal	0.833	0.167	NODE_146_length_78_cov_19
KP28873	case_2	147	chromosomal	0.583	0.417	NODE_147_length_78_cov_72

KP28873	case_2	15	chromosomal	0.982	0.018	NODE_15_length_126807_cov_24.1097
KP28873	case_2	16	chromosomal	0.962	0.038	NODE_16_length_126338_cov_20.7626
KP28873	case_2	17	chromosomal	0.992	0.008	NODE_17_length_108526_cov_21.4616
KP28873	case_2	18	chromosomal	0.996	0.004	NODE_18_length_105981_cov_24.7089
KP28873	case_2	19	chromosomal	0.629	0.371	NODE_19_length_104776_cov_21.3949
KP28873	case_2	2	chromosomal	0.998	0.002	NODE_2_length_292612_cov_23.6881
KP28873	case_2	20	chromosomal	0.996	0.004	NODE_20_length_100509_cov_26.3039
KP28873	case_2	21	chromosomal	0.986	0.014	NODE_21_length_99297_cov_25.1373
KP28873	case_2	22	chromosomal	0.999	0.001	NODE_22_length_94236_cov_25.7175
KP28873	case_2	23	chromosomal	0.975	0.025	NODE_23_length_90092_cov_24.1148
KP28873	case_2	24	chromosomal	0.991	0.009	NODE_24_length_88599_cov_25.2866
KP28873	case_2	25	chromosomal	1	0	NODE_25_length_86605_cov_24.9395
KP28873	case_2	26	chromosomal	0.981	0.019	NODE_26_length_85248_cov_25.2298
KP28873	case_2	27	chromosomal	0.965	0.035	NODE_27_length_84334_cov_20.8779
KP28873	case_2	28	chromosomal	0.981	0.019	NODE_28_length_76440_cov_26.1857
KP28873	case_2	29	chromosomal	0.752	0.248	NODE_29_length_72276_cov_23.3432
KP28873	case_2	3	chromosomal	0.984	0.016	NODE_3_length_232248_cov_20.9202
KP28873	case_2	30	chromosomal	0.962	0.038	NODE_30_length_71671_cov_23.8972
KP28873	case_2	31	chromosomal	0.944	0.056	NODE_31_length_68754_cov_21.9329
KP28873	case_2	32	plasmid	0.012	0.988	NODE_32_length_63961_cov_15.848
KP28873	case_2	33	chromosomal	0.959	0.041	NODE_33_length_62746_cov_23.7741
KP28873	case_2	34	chromosomal	0.987	0.013	NODE_34_length_58972_cov_23.2093
KP28873	case_2	35	chromosomal	0.95	0.05	NODE_35_length_58461_cov_23.7086
KP28873	case_2	36	chromosomal	0.96	0.04	NODE_36_length_49594_cov_23.9551
KP28873	case_2	37	plasmid	0.019	0.981	NODE_37_length_42433_cov_13.9087
KP28873	case_2	38	chromosomal	0.874	0.126	NODE_38_length_40271_cov_21.0941
KP28873	case_2	39	plasmid	0.133	0.867	NODE_39_length_37143_cov_14.4745
KP28873	case_2	4	chromosomal	1	0	NODE_4_length_230166_cov_22.0558
KP28873	case_2	40	chromosomal	0.997	0.003	NODE_40_length_34549_cov_26.4556
KP28873	case_2	41	chromosomal	0.77	0.23	NODE_41_length_34470_cov_22.0205
KP28873	case_2	42	chromosomal	0.909	0.091	NODE_42_length_33910_cov_22.6375
KP28873	case_2	43	chromosomal	0.645	0.355	NODE_43_length_32801_cov_22.1719
KP28873	case_2	44	chromosomal	0.934	0.066	NODE_44_length_29764_cov_24.4033
KP28873	case_2	45	chromosomal	0.944	0.056	NODE_45_length_28468_cov_23.9624
KP28873	case_2	46	chromosomal	0.994	0.006	NODE_46_length_26346_cov_26.3913
KP28873	case_2	47	chromosomal	0.898	0.102	NODE_47_length_26159_cov_21.7188
KP28873	case_2	48	chromosomal	0.768	0.232	NODE_48_length_24358_cov_20.5077
KP28873	case_2	49	chromosomal	0.665	0.335	NODE_49_length_23195_cov_25.9969
KP28873	case_2	5	chromosomal	0.996	0.004	NODE_5_length_210342_cov_20.2479
KP28873	case_2	50	chromosomal	0.593	0.407	NODE_50_length_23028_cov_24.0916
KP28873	case_2	51	chromosomal	0.951	0.049	NODE_51_length_20303_cov_26.2921
KP28873	case_2	52	chromosomal	0.887	0.113	NODE_52_length_19654_cov_24.193
KP28873	case_2	53	chromosomal	0.867	0.133	NODE_53_length_19046_cov_23.7692
KP28873	case_2	54	plasmid	0.436	0.564	NODE_54_length_14967_cov_22.6888
KP28873	case_2	55	chromosomal	0.895	0.105	NODE_55_length_13789_cov_20.5869
KP28873	case_2	56	plasmid	0.081	0.919	NODE_56_length_11614_cov_14.9374
KP28873	case_2	57	plasmid	0.107	0.893	NODE_57_length_8499_cov_12.848
KP28873	case_2	58	chromosomal	0.853	0.147	NODE_58_length_7896_cov_23.201
KP28873	case_2	59	plasmid	0.136	0.864	NODE_59_length_7362_cov_14.3065
KP28873	case_2	6	chromosomal	0.999	0.001	NODE_6_length_203642_cov_23.1249
KP28873	case_2	60	chromosomal	0.892	0.108	NODE_60_length_7357_cov_25.5636
KP28873	case_2	61	plasmid	0.079	0.921	NODE_61_length_7303_cov_11.0559
KP28873	case_2	62	plasmid	0.13	0.87	NODE_62_length_6896_cov_15.4263
KP28873	case_2	63	plasmid	0.026	0.974	NODE_63_length_6424_cov_15.2625
KP28873	case_2	64	plasmid	0.239	0.761	NODE_64_length_6167_cov_19.0051
KP28873	case_2	65	plasmid	0.037	0.963	NODE_65_length_5212_cov_21.6952

KP28873	case_2	66	chromosomal	0.839	0.161	NODE_66_length_4731_cov_20.3616
KP28873	case_2	67	plasmid	0.14	0.86	NODE_67_length_4667_cov_12.3839
KP28873	case_2	68	plasmid	0.099	0.901	NODE_68_length_4510_cov_10.5267
KP28873	case_2	69	plasmid	0.481	0.519	NODE_69_length_4504_cov_14.6869
KP28873	case_2	7	chromosomal	0.994	0.006	NODE_7_length_190612_cov_25.6666
KP28873	case_2	70	plasmid	0.129	0.871	NODE_70_length_3827_cov_34.1757
KP28873	case_2	71	chromosomal	0.855	0.145	NODE_71_length_3823_cov_25.3526
KP28873	case_2	72	chromosomal	0.781	0.219	NODE_72_length_3299_cov_23.4863
KP28873	case_2	73	chromosomal	0.976	0.024	NODE_73_length_3231_cov_240.086
KP28873	case_2	74	plasmid	0.26	0.74	NODE_74_length_2909_cov_16.2479
KP28873	case_2	75	plasmid	0.397	0.603	NODE_75_length_2751_cov_13.0079
KP28873	case_2	76	plasmid	0.198	0.802	NODE_76_length_2615_cov_14.184
KP28873	case_2	77	chromosomal	0.645	0.355	NODE_77_length_2565_cov_10.3593
KP28873	case_2	78	chromosomal	0.64	0.36	NODE_78_length_2438_cov_23.4312
KP28873	case_2	79	plasmid	0.491	0.509	NODE_79_length_2263_cov_16.6102
KP28873	case_2	8	chromosomal	0.997	0.003	NODE_8_length_177371_cov_24.7878
KP28873	case_2	80	plasmid	0.139	0.861	NODE_80_length_1915_cov_135.554
KP28873	case_2	81	plasmid	0.197	0.803	NODE_81_length_1912_cov_15.3292
KP28873	case_2	82	chromosomal	0.983	0.017	NODE_82_length_1888_cov_42.6052
KP28873	case_2	83	chromosomal	0.984	0.016	NODE_83_length_1711_cov_237.209
KP28873	case_2	84	plasmid	0.291	0.709	NODE_84_length_1632_cov_10.2695
KP28873	case_2	85	chromosomal	0.761	0.239	NODE_85_length_1531_cov_39.2221
KP28873	case_2	86	chromosomal	0.999	0.001	NODE_86_length_1445_cov_95.0972
KP28873	case_2	87	plasmid	0.016	0.984	NODE_87_length_1356_cov_25.154
KP28873	case_2	88	plasmid	0.088	0.912	NODE_88_length_1221_cov_260.223
KP28873	case_2	89	plasmid	0.357	0.643	NODE_89_length_1196_cov_199.941
KP28873	case_2	9	chromosomal	0.998	0.002	NODE_9_length_174527_cov_22.6858
KP28873	case_2	90	chromosomal	0.794	0.206	NODE_90_length_1173_cov_45.0465
KP28873	case_2	91	chromosomal	0.506	0.494	NODE_91_length_1169_cov_9.91484
KP28873	case_2	92	chromosomal	0.848	0.152	NODE_92_length_1096_cov_31.4779
KP28873	case_2	93	plasmid	0.131	0.869	NODE_93_length_909_cov_14.929
KP28873	case_2	94	plasmid	0.231	0.769	NODE_94_length_1060_cov_45.5117
KP28873	case_2	95	chromosomal	0.996	0.004	NODE_95_length_1013_cov_53.4893
KP28873	case_2	96	plasmid	0	1	NODE_96_length_820_cov_102.855
KP28873	case_2	97	chromosomal	0.647	0.353	NODE_97_length_669_cov_27.049
KP28873	case_2	98	chromosomal	0.657	0.343	NODE_98_length_661_cov_11.1404
KP28873	case_2	99	plasmid	0.473	0.527	NODE_99_length_607_cov_38.2604

Table S3: Virulomes of *Klebsiella pneumoniae* isolates from this study detected using the Institut Pasteur MLST databases.

Virulence category	Virulence system	Virulence gene	Case	Genome name	ContigID	Pident	Length	Mismatch	Gapopen	Qstart	Qend	Sstart	Send	Eevalue	Bitscore	Coverage	Prediction
**Iron acquisition: Siderophore based uptake systems**	**Fep-Ent System: Enterobactin synthesis**	**entA**	case_1	KP28872	NODE_15_length_126807_cov_40.3171	99.746	786	2	0	1	786	81996	81211	0.0	1441	100	chromosomal
		**entB**	case_1	KP28872	NODE_15_length_126807_cov_40.3171	99.531	852	4	0	1	852	82817	81966	0.0	1552	100	chromosomal
		**entC**	case_1	KP28872	NODE_15_length_126807_cov_40.3171	99.495	1188	6	0	1	1188	85635	84448	0.0	2161	100	chromosomal
		**entD**	case_1	KP28872	NODE_15_length_126807_cov_40.3171	99.683	630	2	0	1	630	99007	99636	0.0	1153	100	chromosomal
		**entE**	case_1	KP28872	NODE_15_length_126807_cov_40.3171	98.632	1608	22	0	1	1608	84438	82831	0.0	2848	100	chromosomal
		**entF**	case_1	KP28872	NODE_15_length_126807_cov_40.3171	99.304	3882	27	0	1	3882	95002	91121	0.0	7020	100	chromosomal
	**Fep-Ent System: Enterobactin receptor**	**fepA**	case_1	KP28872	NODE_15_length_126807_cov_40.3171	99.192	2229	18	0	1	2229	96712	98940	0.0	4017	100	chromosomal
		**fepB**	case_1	KP28872	NODE_15_length_126807_cov_40.3171	99.583	960	4	0	1	960	85812	86771	0.0	1751	100	chromosomal
		**fepC**	case_1	KP28872	NODE_15_length_126807_cov_40.3171	99.245	795	6	0	1	795	90262	91056	0.0	1435	100	chromosomal
		**fepD**	case_1	KP28872	NODE_15_length_126807_cov_40.3171	99.702	1008	3	0	1	1008	88269	89276	0.0	1845	100	chromosomal
		**fepG**	case_1	KP28872	NODE_15_length_126807_cov_40.3171	98.892	993	11	0	1	993	89273	90265	0.0	1773	100	chromosomal
		**fes**	case_1	KP28872	NODE_15_length_126807_cov_40.3171	98.925	1209	13	0	1	1209	96452	95244	0.0	2161	100	chromosomal
		**ybdA**	case_1	KP28872	NODE_15_length_126807_cov_40.3171	99.114	1242	11	0	1	1242	88156	86915	0.0	2233	100	chromosomal

	**IroA System: Salmochelin synthesis**	**iroE**	case_1	KP28872	NODE_1_length_389262_cov_34.9217	99.145	936	8	0	1	936	303158	304093	0.0	1685	100	chromosomal
	**High-pathogenicity island: Yersiniabactin synthesis**	**ybtA**	case_1	KP28872	NODE_50_length_19359_cov_4.40763	99.583	960	4	0	1	960	16122	15163	0.0	1751	100	chromosomal
		**ybtE**	case_1	KP28872	NODE_62_length_10267_cov_4.3949	99.747	1578	4	0	1	1578	7644	6067	0.0	2892	100	plasmid
		**ybtP**	case_1	KP28872	NODE_50_length_19359_cov_4.40763	99.723	1803	5	0	1	1803	16289	18091	0.0	3302	100	chromosomal
		**ybtS**	case_1	KP28872	NODE_130_length_3021_cov_4.33356	97.360	1212	32	0	1	1212	1810	3021	0.0	2061	93	chromosomal
		**ybtT**	case_1	KP28872	NODE_62_length_10267_cov_4.3949	99.627	804	3	0	1	804	8451	7648	0.0	1469	100	plasmid
		**ybtU**	case_1	KP28872	NODE_62_length_10267_cov_4.3949	99.311	1161	8	0	1	1161	9608	8448	0.0	2100	100	plasmid
		**ybtX**	case_1	KP28872	NODE_130_length_3021_cov_4.33356	97.792	1404	31	0	1	1404	379	1782	0.0	2422	100	chromosomal
		**irp1**	case_1	KP28872	NODE_50_length_19359_cov_4.40763	99.715	8777	25	0	1	8777	8777	1	0.0	16070	92	chromosomal
		**irp2**	case_1	KP28872	NODE_50_length_19359_cov_4.40763	99.722	6108	17	0	1	6108	14972	8865	0.0	11186	100	chromosomal
	**High-pathogenicity island: Yersiniabactin receptor**	**fyuA**	case_1	KP28872	NODE_62_length_10267_cov_4.3949	99.753	2022	5	0	1	2022	5936	3915	0.0	3707	100	plasmid
**Fimbriae**	**Type 1 fimbriae**	**fimA**	case_1	KP28872	NODE_9_length_177480_cov_42.0453	99.454	549	3	0	1	549	65635	65087	0.0	998	100	chromosomal
		**fimB**	case_1	KP28872	NODE_9_length_177480_cov_42.0453	99.340	606	4	0	1	606	67795	67190	0.0	1098	100	chromosomal

		**fimC**	case_1	KP28872	NODE_9_length_177480_cov_42.0453	99.862	726	1	0	1	726	64451	63726	0.0	1336	100	chromosomal
		**fimD**	case_1	KP28872	NODE_9_length_177480_cov_42.0453	99.622	2646	10	0	1	2646	63677	61032	0.0	4831	100	chromosomal
		**fimE**	case_1	KP28872	NODE_9_length_177480_cov_42.0453	98.686	609	8	0	1	609	66724	66116	0.0	1081	100	chromosomal
		**fimF**	case_1	KP28872	NODE_9_length_177480_cov_42.0453	99.058	531	5	0	1	531	61024	60494	0.0	953	100	chromosomal
		**fimG**	case_1	KP28872	NODE_9_length_177480_cov_42.0453	99.601	501	2	0	1	501	60481	59981	0.0	915	100	chromosomal
		**fimH**	case_1	KP28872	NODE_9_length_177480_cov_42.0453	99.230	909	7	0	1	909	59966	59058	0.0	1640	100	chromosomal
		**fimI**	case_1	KP28872	NODE_9_length_177480_cov_42.0453	99.371	636	4	0	1	636	65115	64480	0.0	1153	100	chromosomal
		**fimK**	case_1	KP28872	NODE_9_length_177480_cov_42.0453	98.873	1242	14	0	1	1242	58890	57649	0.0	2217	100	chromosomal
	**Type 3 fimbriae**	**mrkA**	case_1	KP28872	NODE_9_length_177480_cov_42.0453	99.836	609	1	0	1	609	72460	73068	0.0	1120	100	chromosomal
		**mrkB**	case_1	KP28872	NODE_9_length_177480_cov_42.0453	99.858	702	1	0	1	702	73164	73865	0.0	1291	100	chromosomal
		**mrkC**	case_1	KP28872	NODE_9_length_177480_cov_42.0453	99.759	2487	6	0	1	2487	73877	76363	0.0	4560	100	chromosomal
		**mrkD**	case_1	KP28872	NODE_9_length_177480_cov_42.0453	99.900	996	1	0	1	996	76354	77349	0.0	1834	100	chromosomal
		**mrkF**	case_1	KP28872	NODE_9_length_177480_cov_42.0453	99.843	636	1	0	1	636	77363	77998	0.0	1170	100	chromosomal
		**mrkH**	case_1	KP28872	NODE_9_length_177480_cov_42.0453	99.719	711	2	0	1	711	80193	79483	0.0	1303	100	chromosomal

		**mrkI**	case_1	KP28872	NODE_9_length_177480_cov_42.0453	99.487	585	3	0	1	585	79477	78893	0.0	1064	100	chromosomal
		**mrkJ**	case_1	KP28872	NODE_9_length_177480_cov_42.0453	99.861	717	1	0	1	717	78749	78033	0.0	1319	100	chromosomal
	**E. coli Common Pilus**	**ecpE (yagV)**	case_1	KP28872	NODE_2_length_292640_cov_38.7891	85.695	755	106	2	3	756	281878	281125	0.0	795	99	chromosomal
		**ecpD (yagW)**	case_1	KP28872	NODE_2_length_292640_cov_38.7891	88.936	1645	180	2	1	1644	283447	281804	0.0	2028	100	chromosomal
		**ecpC (yagX)**	case_1	KP28872	NODE_2_length_292640_cov_38.7891	87.540	2528	311	4	1	2526	285962	283437	0.0	2920	100	chromosomal
		**ecpB (yagY)**	case_1	KP28872	NODE_2_length_292640_cov_38.7891	87.892	669	81	0	1	669	286656	285988	0.0	787	100	chromosomal
		**ecpA (yagZ)**	case_1	KP28872	NODE_2_length_292640_cov_38.7891	90.068	584	58	0	1	584	287302	286719	0.0	758	99	chromosomal
		**ecpR (ykgK)**	case_1	KP28872	NODE_2_length_292640_cov_38.7891	86.403	581	79	0	11	591	287957	287377	0.0	636	98	chromosomal
**Lipopolysaccharide**	**O-antigen: *wb* cluster**	**wzm**	case_1	KP28872	NODE_102_length_3897_cov_4.10759	99.227	776	6	0	5	780	3897	3122	0.0	1400	99	chromosomal
		**wzt**	case_1	KP28872	NODE_102_length_3897_cov_4.10759	99.055	741	7	0	1	741	3122	2382	0.0	1330	100	chromosomal
		**wbbM**	case_1	KP28872	NODE_102_length_3897_cov_4.10759	99.314	1896	13	0	1	1896	2366	471	0.0	3430	100	chromosomal
		**wbbO**	case_1	KP28872	NODE_210_length_1423_cov_3.57727	99.214	1018	8	0	1	1018	1095	78	0.0	1836	90	chromosomal
**Capsule polysaccharide**	**CPS gene loci**	**galF**	case_1	KP28872	NODE_14_length_131869_cov_35.6086	98.885	897	10	0	1	897	38178	39074	0.0	1602	100	chromosomal
		**cpsACP**	case_1	KP28872	NODE_14_length_131869_cov_35.6086	94.436	629	35	0	1	629	39467	40095	0.0	968	99	chromosomal

		**wzi**	case_1	KP28872	NODE_14_length_131869_cov_35.6086	87.574	1521	183	3	1	1515	40975	42495	0.0	1757	100	chromosomal
		**gnd**	case_1	KP28872	NODE_14_length_131869_cov_35.6086	95.665	1407	61	0	1	1407	55441	56847	0.0	2261	100	chromosomal
		**manC**	case_1	KP28872	NODE_124_length_3183_cov_4.47778	98.093	1416	27	0	1	1416	435	1850	0.0	2466	100	chromosomal
		**manB**	case_1	KP28872	NODE_124_length_3183_cov_4.47778	98.551	1311	19	0	1	1311	1873	3183	0.0	2316	96	chromosomal
		**ugd**	case_1	KP28872	NODE_14_length_131869_cov_35.6086	96.829	1167	37	0	1	1167	60702	61868	0.0	1951	100	chromosomal
	**CPS synthesis regulator**	**rcsA**	case_1	KP28872	NODE_5_length_228449_cov_35.4333	100.000	624	0	0	1	624	11111	10488	0.0	1153	100	chromosomal
		**rcsB**	case_1	KP28872	NODE_6_length_203863_cov_36.4848	99.693	651	2	0	1	651	124328	123678	0.0	1192	100	chromosomal
**Outer membrane proteins**	**Outer membrane protein A**	**ompA**	case_1	KP28872	NODE_4_length_230310_cov_35.9925	84.220	1071	139	10	1	1041	133810	134880	0.0	1014	100	chromosomal
	**Efflux pump**	**acrA**	case_1	KP28872	NODE_2_length_292640_cov_38.7891	99.414	1194	7	0	1	1194	128013	129206	0.0	2167	100	chromosomal
		**acrB**	case_1	KP28872	NODE_2_length_292640_cov_38.7891	99.809	3147	6	0	1	3147	129229	132375	0.0	5779	100	chromosomal
**Type VI secretion system (T6SS)**	**Type VI secretion system (T6SS)**	**tssJ (sciN)**	case_1	KP28872	NODE_7_length_203049_cov_34.8131	99.816	543	1	0	1	543	197069	196527	0.0	998	100	chromosomal
		**tssF**	case_1	KP28872	NODE_7_length_203049_cov_34.8131	99.373	1755	11	0	1	1755	199850	198096	0.0	3181	100	chromosomal
		**tssG**	case_1	KP28872	NODE_7_length_203049_cov_34.8131	99.448	1086	6	0	1	1086	198132	197047	0.0	1973	100	chromosomal
		**tssK (vasE)**	case_1	KP28872	NODE_33_length_50217_cov_28.9361	100.000	1344	0	0	1	1344	8133	6790	0.0	2483	100	chromosomal

		**tssB (vipA)**	case_1	KP28872	NODE_33_length_50217_cov_28.9361	99.797	492	1	0	1	492	10221	9730	0.0	904	100	chromosomal
		**tssC (vipB)**	case_1	KP28872	NODE_33_length_50217_cov_28.9361	100.000	1545	0	0	1	1545	9687	8143	0.0	2854	100	chromosomal
		**tssL (dotU)**	case_1	KP28872	NODE_33_length_50217_cov_28.9361	100.000	690	0	0	1	690	6793	6104	0.0	1275	100	chromosomal
		**tssH (clpV)**	case_1	KP28872	NODE_33_length_50217_cov_28.9361	100.000	2655	0	0	1	2655	3640	986	0.0	4903	100	chromosomal
		**tssM (icmF)**	case_1	KP28872	NODE_125_length_3140_cov_31.1877	97.528	3034	75	0	391	3424	3140	107	0.0	5188	89	chromosomal
		**tssA (impA)**	case_1	KP28872	NODE_193_length_1737_cov_3.98253	100.000	380	0	0	29	408	1737	1358	0.0	702	93	chromosomal
		**tssD (hcp)**	case_1	KP28872	NODE_33_length_50217_cov_28.9361	100.000	492	0	0	1	492	4396	3905	0.0	909	100	chromosomal
**PaaR repeat-containing protein**		**KPHS_23120**	case_1	case_1	NODE_111_length_3762_cov_3.86893	100.000	258	0	0	1	258	1844	1587	2.45e-135	477	100	chromosomal
**Cognate immune protein**		**tli1**	case_1	KP28872	NODE_143_length_2713_cov_5.15175	92.697	534	33	4	1	531	643	113	0.0	765	100	chromosomal
**Iron acquisition: Siderophore based uptake systems**	**Fep-Ent System: Enterobactin synthesis**	**entA**	case_2	KP28873	NODE_15_length_126807_cov_24.1097	99.746	786	2	0	1	786	81996	81211	0.0	1441	100	chromosomal
		**entB**	case_2	KP28873	NODE_15_length_126807_cov_24.1097	99.531	852	4	0	1	852	82817	81966	0.0	1552	100	chromosomal
		**entC**	case_2	KP28873	NODE_15_length_126807_cov_24.1097	99.495	1188	6	0	1	1188	85635	84448	0.0	2161	100	chromosomal
		**entD**	case_2	KP28873	NODE_15_length_126807_cov_24.1097	99.683	630	2	0	1	630	99007	99636	0.0	1153	100	chromosomal
		**entE**	case_2	KP28873	NODE_15_length_126807_cov_24.1097	98.632	1608	22	0	1	1608	84438	82831	0.0	2848	100	chromosomal

		**entF**	case_2	KP28873	NODE_15_length_126807_cov_24.1097	99.304	3882	27	0	1	3882	95002	91121	0.0	7020	100	chromosomal
	**Fep-Ent System: Enterobactin receptor**	**fepA**	case_2	KP28873	NODE_15_length_126807_cov_24.1097	99.192	2229	18	0	1	2229	96712	98940	0.0	4017	100	chromosomal
		**fepB**	case_2	KP28873	NODE_15_length_126807_cov_24.1097	99.583	960	4	0	1	960	85812	86771	0.0	1751	100	chromosomal
		**fepC**	case_2	KP28873	NODE_15_length_126807_cov_24.1097	99.245	795	6	0	1	795	90262	91056	0.0	1435	100	chromosomal
		**fepD**	case_2	KP28873	NODE_15_length_126807_cov_24.1097	99.702	1008	3	0	1	1008	88269	89276	0.0	1845	100	chromosomal
		**fepG**	case_2	KP28873	NODE_15_length_126807_cov_24.1097	98.892	993	11	0	1	993	89273	90265	0.0	1773	100	chromosomal
		**fes**	case_2	KP28873	NODE_15_length_126807_cov_24.1097	98.925	1209	13	0	1	1209	96452	95244	0.0	2161	100	chromosomal
		**ybdA**	case_2	KP28873	NODE_15_length_126807_cov_24.1097	99.114	1242	11	0	1	1242	88156	86915	0.0	2233	100	chromosomal
	**IroA System: Salmochelin synthesis**	**iroE**	case_2	KP28873	NODE_3_length_232248_cov_20.9202	99.145	936	8	0	1	936	86105	85170	0.0	1685	100	chromosomal
**Fimbriae**	**Type 1 fimbriae**	**fimA**	case_2	KP28873	NODE_8_length_177371_cov_24.7878	99.454	549	3	0	1	549	65635	65087	0.0	998	100	chromosomal
		**fimB**	case_2	KP28873	NODE_8_length_177371_cov_24.7878	99.340	606	4	0	1	606	67795	67190	0.0	1098	100	chromosomal
		**fimC**	case_2	KP28873	NODE_8_length_177371_cov_24.7878	99.862	726	1	0	1	726	64451	63726	0.0	1336	100	chromosomal
		**fimD**	case_2	KP28873	NODE_8_length_177371_cov_24.7878	99.622	2646	10	0	1	2646	63677	61032	0.0	4831	100	chromosomal
		**fimE**	case_2	KP2887	NODE_8_length_177371_cov_24.7878	98.686	609	8	0	1	609	66724	66116	0.0	1081	100	chromosomal

				3													
		**fimF**	case_2	KP28873	NODE_8_length_177371_cov_24.7878	99.058	531	5	0	1	531	61024	60494	0.0	953	100	chromosomal
		**fimG**	case_2	KP28873	NODE_8_length_177371_cov_24.7878	99.401	501	3	0	1	501	60481	59981	0.0	909	100	chromosomal
		**fimH**	case_2	KP28873	NODE_8_length_177371_cov_24.7878	99.230	909	7	0	1	909	59966	59058	0.0	1640	100	chromosomal
		**fimI**	case_2	KP28873	NODE_8_length_177371_cov_24.7878	99.371	636	4	0	1	636	65115	64480	0.0	1153	100	chromosomal
		**fimK**	case_2	KP28873	NODE_8_length_177371_cov_24.7878	98.873	1242	14	0	1	1242	58890	57649	0.0	2217	100	chromosomal
	**Type 3 fimbriae**	**mrkA**	case_2	KP28873	NODE_8_length_177371_cov_24.7878	99.836	609	1	0	1	609	72460	73068	0.0	1120	100	chromosomal
		**mrkB**	case_2	KP28873	NODE_8_length_177371_cov_24.7878	99.858	702	1	0	1	702	73164	73865	0.0	1291	100	chromosomal
		**mrkC**	case_2	KP28873	NODE_8_length_177371_cov_24.7878	99.759	2487	6	0	1	2487	73877	76363	0.0	4560	100	chromosomal
		**mrkD**	case_2	KP28873	NODE_8_length_177371_cov_24.7878	99.900	996	1	0	1	996	76354	77349	0.0	1834	100	chromosomal
		**mrkF**	case_2	KP28873	NODE_8_length_177371_cov_24.7878	99.843	636	1	0	1	636	77363	77998	0.0	1170	100	chromosomal
		**mrkH**	case_2	KP28873	NODE_8_length_177371_cov_24.7878	99.719	711	2	0	1	711	80193	79483	0.0	1303	100	chromosomal
		**mrkI**	case_2	KP28873	NODE_8_length_177371_cov_24.7878	99.487	585	3	0	1	585	79477	78893	0.0	1064	100	chromosomal
		**mrkJ**	case_2	KP28873	NODE_8_length_177371_cov_24.7878	99.861	717	1	0	1	717	78749	78033	0.0	1319	100	chromosomal
	**E. coli Common**	**ecpE (yagV)**	case_2	KP2887	NODE_2_length_292612_cov_23.6881	85.695	755	106	2	3	756	281738	280985	0.0	795	99	chromosomal

	**Pilus**			3													
		**ecpD (yagW)**	case_2	KP28873	NODE_2_length_292612_cov_23.6881	88.936	1645	180	2	1	1644	283307	281664	0.0	2028	100	chromosomal
		**ecpC (yagX)**	case_2	KP28873	NODE_2_length_292612_cov_23.6881	87.540	2528	311	4	1	2526	285822	283297	0.0	2920	100	chromosomal
		**ecpB (yagY)**	case_2	KP28873	NODE_2_length_292612_cov_23.6881	87.892	669	81	0	1	669	286516	285848	0.0	787	100	chromosomal
		**ecpA (yagZ)**	case_2	KP28873	NODE_2_length_292612_cov_23.6881	90.068	584	58	0	1	584	287162	286579	0.0	758	99	chromosomal
		**ecpR (ykgK)**	case_2	KP28873	NODE_2_length_292612_cov_23.6881	86.403	581	79	0	11	591	287817	287237	0.0	636	98	chromosomal
**Capsule polysaccharide**	**CPS gene loci**	**galF**	case_2	KP28873	NODE_29_length_72276_cov_23.3432	98.885	897	10	0	1	897	34325	33429	0.0	1602	100	chromosomal
		**cpsACP**	case_2	KP28873	NODE_29_length_72276_cov_23.3432	94.436	629	35	0	1	629	33036	32408	0.0	968	99	chromosomal
		**wzi**	case_2	KP28873	NODE_29_length_72276_cov_23.3432	87.574	1521	183	3	1	1515	31528	30008	0.0	1757	100	chromosomal
		**gnd**	case_2	KP28873	NODE_29_length_72276_cov_23.3432	95.665	1407	61	0	1	1407	17062	15656	0.0	2261	100	chromosomal
		**ugd**	case_2	KP28873	NODE_29_length_72276_cov_23.3432	96.829	1167	37	0	1	1167	11801	10635	0.0	1951	100	chromosomal
	**CPS synthesis regulator**	**rcsA**	case_2	KP28873	NODE_31_length_68754_cov_21.9329	100.000	624	0	0	1	624	57644	58267	0.0	1153	100	chromosomal
		**rcsB**	case_2	KP28873	NODE_6_length_203642_cov_23.1249	99.693	651	2	0	1	651	124107	123457	0.0	1192	100	chromosomal
**Outer membrane proteins**	**Outer membrane protein A**	**ompA**	case_2	KP28873	NODE_4_length_230166_cov_22.0558	84.220	1071	139	10	1	1041	96357	95287	0.0	1014	100	chromosomal
	**Efflux pump**	**acrA**	case_	KP2887	NODE_2_length_292612_cov_23.6	99.414	1194	7	0	1	1194	12787	12906	0.0	2167	100	chromosom

			2	3	881							3	6				al
		**acrB**	case_2	KP28873	NODE_2_length_292612_cov_23.6881	99.809	3147	6	0	1	3147	129089	132235	0.0	5779	100	chromosomal
**Type VI secretion system (T6SS)**	**Type VI secretion system (T6SS)**	**tssM (icmF)**	case_2	KP28873	NODE_5_length_210342_cov_20.2479	97.403	3080	80	0	345	3424	4185	7264	0.0	5245	90	chromosomal
		**tssJ (sciN)**	case_2	KP28873	NODE_5_length_210342_cov_20.2479	99.816	543	1	0	1	543	13274	13816	0.0	998	100	chromosomal
		**tssF**	case_2	KP28873	NODE_5_length_210342_cov_20.2479	99.373	1755	11	0	1	1755	10493	12247	0.0	3181	100	chromosomal
		**tssG**	case_2	KP28873	NODE_5_length_210342_cov_20.2479	99.448	1086	6	0	1	1086	12211	13296	0.0	1973	100	chromosomal

Table S4: Antimicrobial resistance genes detected using ResFinder 3.1 from *K. pneumoniae* isolates from this study.

Case	Genome name	Database	Resistance gene	Identity	Query / Template length	ContigID	Position in contig	Predicted phenotype	Accession number	Prediction
case_1	KP28872	aminoglycoside and flouroquinolone	aac(6')-Ib-cr	100	600 / 600	NODE_133_length_2976_cov_15.4984	2264..2863	Fluoroquinolone and aminoglycoside resistance	DQ303918	plasmid
case_1	KP28872	aminoglycoside	aadA1	100	792 / 792	NODE_84_length_5212_cov_29.0715	1825..2616	Aminoglycoside resistance	JX185132	plasmid
case_1	KP28872	aminoglycoside	aadA16	99.65	846 / 846	NODE_133_length_2976_cov_15.4984	83..928	Aminoglycoside resistance	EU675686	plasmid
case_1	KP28872	aminoglycoside	aph(3'')-Ib	100	804 / 804	NODE_52_length_16943_cov_14.2677	9480..10283	Aminoglycoside resistance Alternate name; aph(3'')-Ib	AF321551	plasmid
case_1	KP28872	aminoglycoside	aph(6)-Id	100	837 / 837	NODE_52_length_16943_cov_14.2677	10283..11119	Aminoglycoside resistance Alternate name; aph(6)-Id	M28829	plasmid
case_1	KP28872	beta-lactam (ESBL)	blaCTX-M-15	100	876 / 876	NODE_52_length_16943_cov_14.2677	15522..16397	Beta-lactam resistance Alternate name; UOE-1	AY044436	plasmid
case_1	KP28872	beta-lactam	blaOXA-1	100	831 / 831	NODE_199_length_1632_cov_11.409	657..1487	Beta-lactam resistance	HQ170510	plasmid
case_1	KP28872	beta-lactam	blaSHV-187	99.88	867 / 867	NODE_1_length_389262_cov_34.9217	381172..382038	Beta-lactam resistance	LN515533	chromosomal
case_1	KP28872	beta-lactam	blaTEM-1B	100	861 / 861	NODE_52_length_16943_cov_14.2677	11840..12700	Beta-lactam resistance Alternate name; RblaTEM-1	AY458016	plasmid
case_1	KP28872	fosfomycin	fosA	99.29	420 / 420	NODE_3_length_260172_cov_39.2366	14164..14583	Fosfomycin resistance	ACWO01000079	chromosomal
case_1	KP28872	phenicol	catA1	99.85	660 / 660	NODE_189_length_1787_cov_15.7918	428..1087	Phenicol resistance	V00622	plasmid
case_1	KP28872	phenicol	catB3	100	442 / 633	NODE_199_length_1632_cov_11.409	78..519	Phenicol resistance	AJ009818	plasmid
case_1	KP28872	quinolone	oqxA	100	1176 / 1176	NODE_31_length_62746_cov_96.3854	3669..4844	Quinolone resistance	EU370913	chromosomal
case_1	KP28872	quinolone	oqxB	99.27	3153 / 3153	NODE_31_length_62746_cov_96.3854	4868..8020	Quinolone resistance	EU370913	chromosomal
case_1	KP28872	rifampicin	ARR-3	100	453 / 453	NODE_133_length_2976_cov_15.4984	1715..2167	Rifampicin resistance	JF806499	plasmid
case_1	KP28872	sulphonamide	sul1	100	840 / 840	NODE_84_length_5212_cov_29.0715	481..1320	Sulphonamide resistance	U12338	plasmid
case_1	KP28872	sulphonamide	sul2	100	816 / 816	NODE_52_length_16943_cov_14.2677	8604..9419	Sulphonamide resistance	AY034138	plasmid
case_1	KP28872	trimethoprim	dfrA1	99.79	474 / 474	NODE_84_length_5212_cov_29.0715	2709..3182	Trimethoprim resistance	AF203818	plasmid
case_1	KP28872	trimethoprim	dfrA14	100	474 / 474	NODE_76_length_6551_cov_10.9166	5988..6461	Trimethoprim resistance	KF921535	plasmid
case_1	KP28872	trimethoprim	dfrA27	100	474 / 474	NODE_133_length_2976_cov_15.4984	1109..1582	Trimethoprim resistance	FJ459817	plasmid
case_2	KP28873	aminoglycoside and flouroquinolone	aac(6')-Ib-cr	100	600 / 600	NODE_74_length_2909_cov_16.2479	2264..2863	Fluoroquinolone and aminoglycoside resistance	DQ303918	plasmid
case_2	KP28873	aminoglycoside	aadA1	100	792 / 792	NODE_65_length_5212_cov_21.6952	1825..2616	Aminoglycoside resistance	JX185132	plasmid
case_2	KP28873	aminoglycoside	aadA16	99.65	846 / 846	NODE_74_length_2909_cov_16.2479	83..928	Aminoglycoside resistance	EU675686	plasmid
case_2	KP28873	aminoglycoside	aph(3'')-Ib	100	804 / 804	NODE_61_length_7303_cov_11.0559	5119..5922	Aminoglycoside resistance Alternate name; aph(3'')-Ib	AF321551	plasmid
case_2	KP28873	aminoglycoside	aph(6)-Id	100	837 / 837	NODE_61_length_7303_cov_11.0559	4283..5119	Aminoglycoside resistance Alternate name; aph(6)-Id	M28829	plasmid
case_2	KP28873	beta-lactam	blaOXA-1	100	831 / 831	NODE_84_length_1632_cov_10.2695	657..1487	Beta-lactam resistance	HQ170510	plasmid

Supplementary Materials

case_2	KP28873	beta-lactam	blaSHV-187	99.88	867 / 867	NODE_3_length_232248_cov_20.9202	7225..8091	Beta-lactam resistance	LN515533	chromosomal
case_2	KP28873	beta-lactam	blaTEM-1B	100	861 / 861	NODE_61_length_7303_cov_11.0559	2702..3562	Beta-lactam resistance Alternate name; RblaTEM-1	AY458016	plasmid
case_2	KP28873	fosfomycin	fosA	99.29	420 / 420	NODE_1_length_355714_cov_24.3492	14164..14583	Fosfomycin resistance	ACWO01000079	chromosomal
case_2	KP28873	phenicol	catA1	99.85	660 / 660	NODE_81_length_1912_cov_15.3292	546..1205	Phenicol resistance	V00622	plasmid
case_2	KP28873	phenicol	catB3	100	442 / 633	NODE_84_length_1632_cov_10.2695	78..519	Phenicol resistance	AJ009818	plasmid
case_2	KP28873	quinolone	oqxA	100	1176 / 1176	NODE_33_length_62746_cov_23.7741	3669..4844	Quinolone resistance	EU370913	chromosomal
case_2	KP28873	quinolone	oqxB	99.27	3153 / 3153	NODE_33_length_62746_cov_23.7741	4868..8020	Quinolone resistance	EU370913	chromosomal
case_2	KP28873	rifampicin	ARR-3	100	453 / 453	NODE_74_length_2909_cov_16.2479	1715..2167	Rifampicin resistance	JF806499	plasmid
case_2	KP28873	sulphonamide	sul1	100	840 / 840	NODE_65_length_5212_cov_21.6952	481..1320	Sulphonamide resistance	U12338	plasmid
case_2	KP28873	sulphonamide	sul2	100	816 / 816	NODE_61_length_7303_cov_11.0559	5983..6798	Sulphonamide resistance	AY034138	plasmid
case_2	KP28873	trimethoprim	dfrA1	99.79	474 / 474	NODE_65_length_5212_cov_21.6952	2709..3182	Trimethoprim resistance	AF203818	plasmid
case_2	KP28873	trimethoprim	dfrA27	100	474 / 474	NODE_74_length_2909_cov_16.2479	1109..1582	Trimethoprim resistance	FJ459817	plasmid

Table S5: The presence of integrative and conjugative element ICEKp1 and ICEKp2 in this study.

Marker genes	Genome name	ContigID	Pident	Length	Mismatch	Gapopen	Qstart	Qend	Sstart	Send	Evalue	Bitscore	Coverage	ICEKp	Case	Prediction
int	KP28872	NODE_69_length_7821_cov_4.72314	97.245	1234	34	0	1	1234	238	1471	0	2091	98	ICEKp1	case_1	chromosomal
irp1	KP28872	NODE_50_length_19359_cov_4.40763	99.656	6108	21	0	1	6108	14972	8865	0	11164	100	ICEKp1	case_1	chromosomal
ybtT	KP28872	NODE_62_length_10267_cov_4.3949	99.729	738	2	0	1	738	8385	7648	0	1352	100	ICEKp1	case_1	plasmid
virB4	KP28872	NODE_85_length_5208_cov_3.69577	97.481	2739	69	0	1	2739	194	2932	0	4676	100	ICEKp1	case_1	plasmid
mobB	KP28872	NODE_163_length_2283_cov_4.06754	92.736	1597	114	2	1	1596	688	2283	0	2305	86	ICEKp1	case_1	chromosomal
int2a	KP28872	NODE_137_length_2875_cov_4.63331	100	801	0	0	1	801	1118	318	0	1480	100	ICEKp2	case_1	chromosomal
int2b	KP28872	NODE_176_length_2015_cov_4.20691	100	808	0	0	1	808	808	1	0	1493	90	ICEKp2	case_1	chromosomal
mcp	KP28872	NODE_110_length_3772_cov_4.08444	100	741	0	0	1	741	843	103	0	1369	100	ICEKp2	case_1	chromosomal
traI	KP28872	NODE_137_length_2875_cov_4.63331	100	1290	0	0	1	1290	2571	1282	0	2383	100	ICEKp2	case_1	chromosomal
mob2	KP28872	NODE_60_length_10594_cov_4.70533	100	2100	0	0	1	2100	8912	6813	0	3879	100	ICEKp2	case_1	chromosomal
traU/PFL_4710	KP28872	NODE_95_length_4393_cov_4.24351	99.894	942	1	0	1	942	2116	3057	0	1735	100	ICEKp2	case_1	chromosomal
	KP28873	-	-	-	-	-	-	-	-	-	-	-	-	negative to ICEKp1 and ICEKp2	case_2	-

Table S6: Plasmid replicons types identified in the study.

Case	Genome name	Plasmid	Identity	Query / Template length	ContigID	Position in contig	Accession number	Prediction
case_1	KP28872	**IncFIB(K)**	100	560 / 560	NODE_77_length_6167_cov_22.7044	3477..4036	JN233704	plasmid
case_1	KP28872	**IncFII(K)**	100	148 / 148	NODE_116_length_3602_cov_15.9452	1741..1888	CP000648	plasmid
case_2	KP28873	**IncFIB(K)**	100	560 / 560	NODE_64_length_6167_cov_19.0051	3477..4036	JN233704	plasmid
case_2	KP28873	**IncFII(K)**	100	148 / 148	NODE_59_length_7362_cov_14.3065	1741..1888	CP000648	plasmid
